# “Phylogenetic and evolutionary analysis of functional divergence among Gamma glutamyl transpeptidase (GGT) subfamilies”

**DOI:** 10.1186/s13062-015-0080-7

**Published:** 2015-09-14

**Authors:** Ved Vrat Verma, Rani Gupta, Manisha Goel

**Affiliations:** Department of Biophysics, University of Delhi, South Campus, New Delhi, 110021 India; Department of Microbiology, University of Delhi, South Campus, New Delhi, 110021 India

**Keywords:** Binding cavity, Positive selection, Protein engineering, Type I functional divergence, Type II functional divergence

## Abstract

**Background:**

γ-glutamyltranspeptidase (GGT) is a bi-substrate enzyme conserved in all three domains of life. It catalyzes the cleavage and transfer of γ-glutamyl moiety of glutathione to either water (hydrolysis) or substrates like peptides (transpeptidation). GGTs exhibit great variability in their enzyme kinetics although the mechanism of catalysis is conserved. Recently, GGT has been shown to be a virulence factor in microbes like *Helicobacter pylori* and *Bacillus anthracis*. In mammalian cells also, GGT inhibition prior to chemotherapy has been shown to sensitize tumors to the therapy. Therefore, lately both bacterial and eukaryotic GGTs have emerged as potential drug targets, but the efforts directed towards finding suitable inhibitors have not yielded any significant results yet. We propose that delineating the residues responsible for the functional diversity associated with these proteins could help in design of species/clade specific inhibitors.

**Results:**

In the present study, we have carried out phylogenetic analysis on a set of 47 GGT-like proteins to address the functional diversity. These proteins segregate into various subfamilies, forming separate clades on the tree. Sequence conservation and motif prediction studies show that even though most of the highly conserved residues have been characterized biochemically in previous studies, a significant number of novel putative sites and motifs are discovered that vary in a clade specific manner. Many of the putative sites predicted during the functional divergence type I and type II analysis, lie close to the known catalytic residues and line the walls of the substrate binding cavity, reinforcing their role in modulating the substrate specificity, catalytic rates and stability of this protein.

**Conclusion:**

The study offers interesting insights into the evolution of GGT-like proteins in pathogenic vs. non-pathogenic bacteria, archaea and eukaryotes. Our analysis delineates residues that are highly specific to each GGT subfamily. We propose that these sites not only explain the differences in stability and catalytic variability of various GGTs but can also aid in design of specific inhibitors against particular GGTs. Thus, apart from the commonly used *in-silico* inhibitor screening approaches, evolutionary analysis identifying the functional divergence hotspots in GGT proteins could augment the structure based drug design approaches.

**Reviewers:**

This article was reviewed by Andrei Osterman, Christine Orengo, and Srikrishna Subramanian. For complete reports, see the Reviewers’ reports section

**Electronic supplementary material:**

The online version of this article (doi:10.1186/s13062-015-0080-7) contains supplementary material, which is available to authorized users.

## Background

Gamma glutamyl transpeptidase (GGT; EC 2.3.2.2) is a bi-substrate enzyme which catalyzes the cleavage of γ-glutamyl linkage of glutathione (GSH) and the transfer of its γ-glutamyl group to acceptor substrates like water (hydrolysis reaction) or other amino acids or peptides (transpeptidation reaction) [1–3]. Evolutionarily, GGT is ubiquitous and conserved in all three domains of life, prokaryotes, eukaryotes and archaea [4]. GGT plays a key role in glutathione metabolism and balances the levels of intracellular cysteine in cells, thus helping in maintenance of the redox equilibrium inside the cells [5, 6]. Other than glutathione metabolism, GGT is also a key enzyme for other metabolic processes like arachidonic acid metabolism, cyanoamino acid metabolism, gamma-glutamyl cycle, hypoglycin biosynthesis, and taurine and hypotaurine metabolism [7–12].

GGT proteins are member of N-terminal nucleophile hydrolase (NTN-hydrolase) superfamily of enzymes. These are heterodimeric proteins, composed of a large and a small subunit, which result from cleavage of a single protein chain through an auto-catalytic processing reaction after synthesis [13–15]. Members of the GGT protein family share a conserved “sandwich like” 3D structural domain which is made up of four layered αββα fold, except in case of CapD protein from *B. anthracis,* which has been reported to have six layered ααββαα like fold. The catalytic site of GGT appears to be formed of two consecutive pockets, the donor and the acceptor sites. The donor site, where the substrate donating γ-glutamyl group binds, has been more extensively characterized so far whereas much less is known about the residues participating in the acceptor site [16]. Some members of the GGT protein family possess an additional flexible loop covering the substrate binding cleft, known as lid-loop region. This lid-loop has been proposed to influence the transpeptidation reaction of GGT proteins [17].

In all GGT proteins characterized so far, a conserved threonine (Thr) acts as nucleophile during it’s auto-processing into small and large subunits as well as during it’s catalysis reaction [1–3]. In the first step of catalysis, the hydroxyl group of Thr attacks the carbonyl group of the glutathione substrate (Additional file 1). The second step is the formation of a transition state. The third step involves the release of ‘Cys-Gly’ from glutathione substrate, leading to the formation of a γ-glutamyl-GGT intermediate complex (Additional file 1). This intermediate complex is stabilized through hydrogen bonds of the substrate with two conserved glycines of GGT, commonly known as “oxyanione hole residues”. The fourth and the final step of this mechanism involves the transfer of the γ-glutamyl moiety to water or short peptide.

A vast variety of work characterizing various GGT homologs from many species has been done in past because of its importance in clinical as well as biotechnological sectors. Clinically, GGT activity in human serum is a common diagnostic indicator of several diseases including liver cancer, alcoholic hepatitis, disrupted bile formation, pancreatic cancer and other hepatic or biliary tract-associated diseases. While GGT deficiency leads to diseases like glutathionemia and glutathionuria associated with mental retardation, its overexpression has been implicated in asthama, parkinson, arthritis and cardiovascular diseases in humans [18–22]. In mammalian cells, GGT inhibition prior to chemotherapy treatments has been shown to sensitize tumours to the therapy [23]. Thus, there are instances where inhibiting GGT activity offers physiological benefits, thus necessitating the need to design inhibitors against GGTs. In microbes, GGT is known to be a virulence factor associated with anchoring the capsule to the bacterial cell wall as well as participating in capsule remodelling in *Bacillus anthracis*. GGT has been also associated with the colonization of the gastric mucosa by *Helicobacter pylori*, the pathogenic bacteria known for ulcer and gastric cancer [24–26]. The CD_4_-positive T cells of the host are crucial for bacterial elimination from the host, which are known to be inhibited by *Helicobacter pylori* GGT, thus promoting the survival of the pathogen [27]. Inhibitors targeting these microbial GGTs may thus complement or augment the effect of currently available antibiotics. Given the above observations, there have been continuous efforts to design inhibitors against both the human as well as the microbial GGTs. The most obvious inhibitors for this enzyme are the donor substrate (glutamate) analogs but these appear to be toxic for human use, leaving the scope open for design of novel GGT inhibitors. Recently, some progress has been reported in this area, with the design of a novel class of species-specific inhibitor (OU749) against GGT, which seems to inhibit human GGT specifically but have no effect on GGTs from closely related species like rat and mice [28]. However, the details of its mode of binding and inhibition are not known in detail yet. Other than its medical significance, GGT also happens to be a biotechnologically useful enzyme [20, 29–34]. The three dimensional structures of GGTs from varied organisms, including human GGT1, *E. coli*, *H. pylori, B. subtilis*, *B. licheniformis*, *B. halodurans* and *T. acidophilum*, with and without substrate analogues, small ions or inhibitors bound to the protein, are now available (Additional file 2) [17, 29–31, 35–37]. Crystal structures are also available for GGT-like protein “CapD” from *B. anthracis* [38]. Comparison of these structures is expected to assist the design of inhibitors against specific GGTs and also help in delineating features responsible for substrate specificity and protein stability, thus providing leads to engineering GGT proteins with desirable biotechnological properties.

In the present study we undertook a comprehensive comparative analysis of 47 GGT-like proteins from all three domains of life. We have looked for conserved motifs and compared the distribution of residues known to be functionally important, which may thereby affect the conservation or diversification of functions in GGT-family proteins. The phylogenetic tree clusters the GGT protein sequences into various clades. Well established statistical methods were used to determine whether GGT genes of specific clades are under positive selection with respect to the background tree. Further, type I and type II divergence analyses were carried out for the GGT clades showing positive selection for the identification of specific divergent sites which might be contributing to the functional divergence. Here, we discuss the potential role of these divergent sites in protein structure and function. Although, experimental characterization may be required for validating the role of these sites, current study shows promise in being able to identify sites that may be responsible for differences in substrate specificities or imparting other valuable functional or physiochemical differences observed in GGT proteins.

## Results and discussion

### Phylogenetic analysis of the GGT proteins

**Fig. 1:**
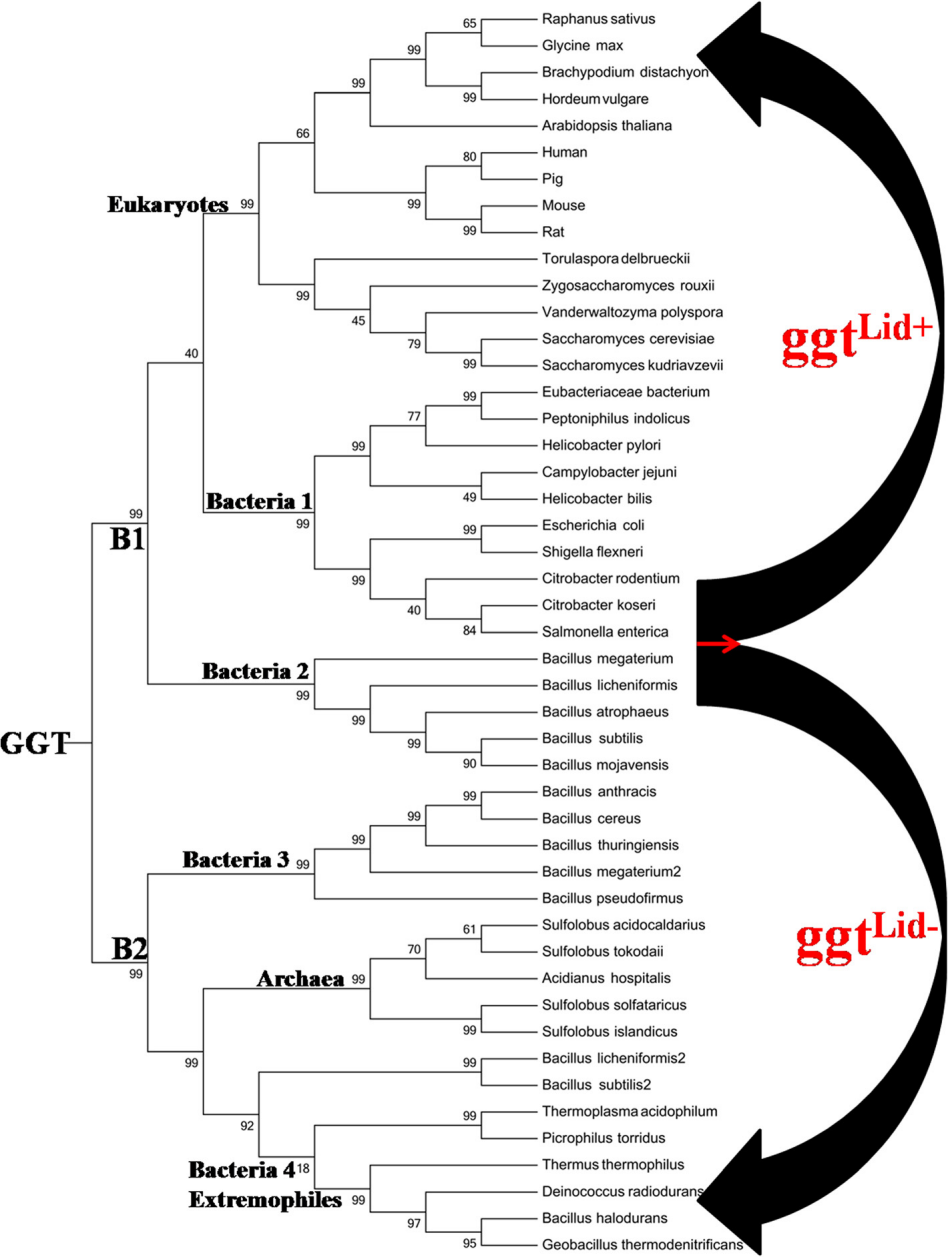
Phylogenetic tree of gamma-glutamyl transpeptidase family. Phylogenetic trees of gamma-glutamyl transpeptidase family were made based on NJ and ML analysis. Major groups of GGT-family resolved in two main branches B1 and B2, further divided into six clades: Eukaryotes, Bacteria (1, 2 and 3), Archaea and Bacteria 4 extremophiles. The eukaryotes and bacteria 1 clade, are further divided into 3 and 2 subfamilies respectively. Further, the same tree is separated into two major groups; **ggt** ^**Lid+**^: GGT sequences containing lid-loop regions and **ggt** ^**Lid-**^: GGT sequences not containing lid-loop regions. The final log likelihood of the ML tree is -21446.23 and the gamma shape parameter is 1.46

The phylogenetic tree of selected 47 GGT sequences divides the tree into two main branches, B1 and B2, which seem to differ from each other mainly by the presence of eukaryotic and archaeal GGT proteins on them, respectively. These clades are referred to as “eukaryotes (Euk)” and “archaeal (Arch)” clades further in the manuscript (Fig. 1). On branch B1, GGT sequences from lower and higher eukaryotes form two subclades within the “eukaryotes (Euk)” clade. The large number of GGT sequences from bacterial organisms cluster into four distinct clades, which are distributed on both branches. Two of the bacterial clades are located on branch B1 along with the eukaryotes clade and are referred to as the “Bacteria 1 (Bact1)” and “Bacteria 2 (Bact2)” clades (Fig. 1). The “Bact1” clade could also be referred to as the “pathogenic clade” since all bacterial species on this clade are known to be infectious to humans. The two other clades of bacterial GGT sequences lie on branch B2, along with the GGT proteins from archaea. One of these is referred to as the “Bacteria 3 (Bact3)” clade, while the other is referred to as the “Bacteria-extremophiles (Bact4ext)” clade (Fig. 1). The clade “Bact4ext” holds GGT sequences from bacterial organisms which are known to inhabit extreme environments.

Such a distribution of GGT proteins on the phylogenetic tree offers several interesting insights into the evolutionary history of GGT proteins. The “Bact1” clade, exclusively composed of bacterial organisms known to be pathogenic for human, appears to be more closely related to the eukaryotic clade “Euk” than to the “Bact2” clade on the same branch (B1). This implies that the host and pathogenic organisms share more similarities in their GGT protein sequences than these pathogenic bacteria do with other bacterial GGTs. Also, in case of some organisms like *B. licheniformis* and *B. subtilis* which code for two GGT-like proteins, the two proteins are distantly placed on two separate branches B1 and B2, unlike what is expected from paralogs (Fig. 1). While one GGT protein from each organism is part of the “Bact2” clade on branch B1, and the other GGT-like protein clusters with the extremophiles “Bact4ext” clade on branch B2. This suggests that either the two GGT proteins in these organisms have diverged quite a long time back and have acquired vastly different functions or one of the proteins is a canonical GGT, while the other has been introduced into the ancestors of *B. subtilis/B. licheniformis* through horizontal gene transfer from some other archaeal or extremophilic organisms. Since most archaeal organisms naturally inhabit in extreme environmental conditions like low pH or/and high temperature conditions, it is not surprising that the “Arch” clade holding GGT sequences from such archaeal organisms are most closely related to the “Bact4ext” clade.

Interestingly, the phylogenetic tree also resolved the GGT proteins into two distinct groups: the first group comprises of GGT sequences having the lid-loop fragment (ggt^Lid+^) and the other group includes GGT sequences missing the lid-loop fragment (ggt^Lid-^) (Fig. 1). This lid-loop fragment is a small stretch of about 12 amino acids in some GGT proteins, which makes a lid like structure covering the opening of the substrate binding cavity of respective proteins. This lid-loop is present in GGTs from *E. coli* (Pro438-Gly449), *H. pylori* (Pro427-Gly438) and “Euk” (human; Ser428-Ser438) but absent from GGTs comprising the “Bact4ext” (*B. haloduran*), “Arch” (*T. acidophilum, S. solfataricus*), “Bact3” (CapD of *B. anthracis*) and “Bact2” (*B. subtilis*) clusters (Additional file 3). The presence of this lid has earlier been suggested to enhance the transpeptidase activity in GGT proteins [18].

### Conserved motifs and functionally important residues in GGT proteins

**Fig. 2:**
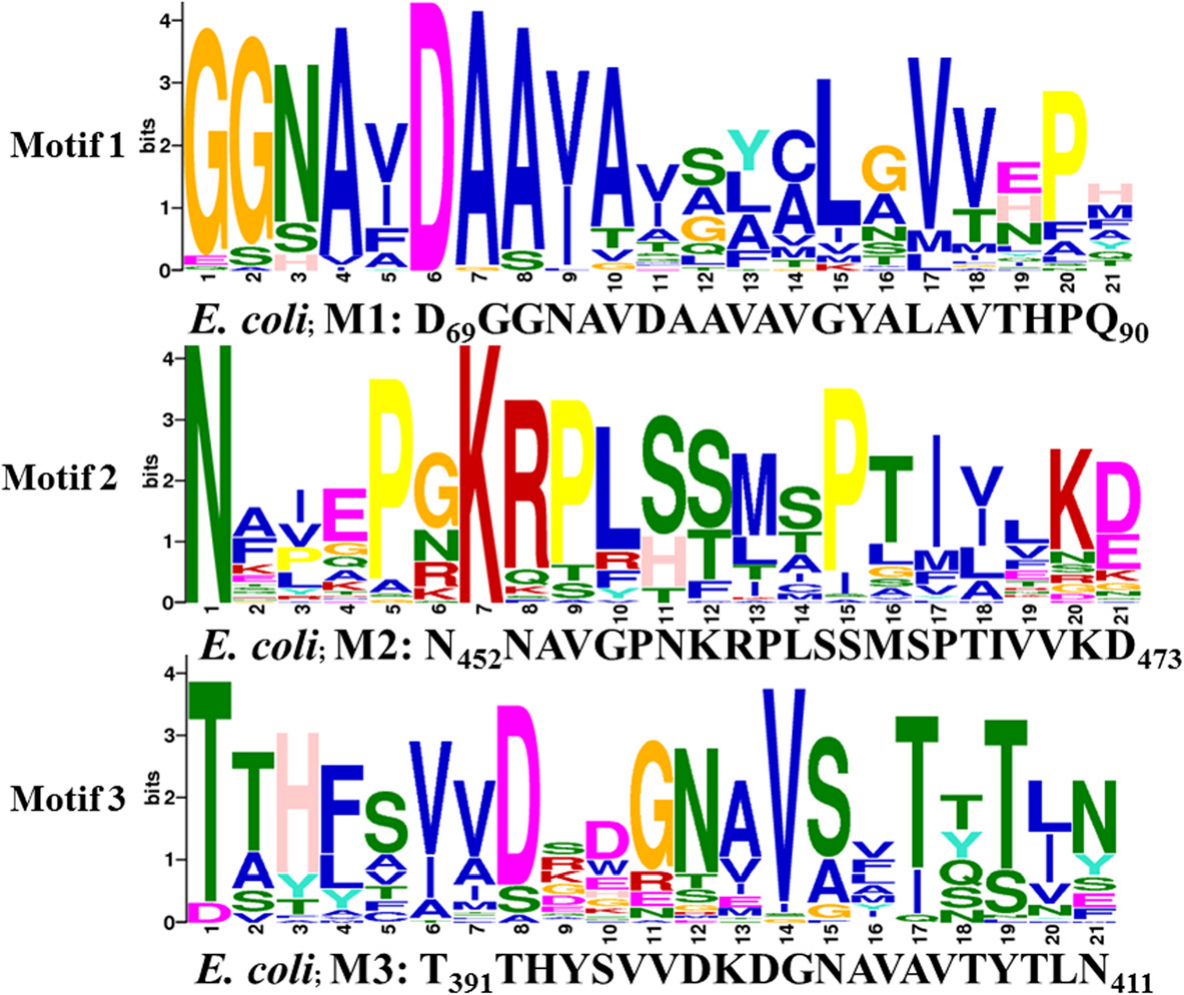
Functional motif analysis in GGT family. Figure shows three putative motifs M1, M2 and M3 identified by MEME in the GGT alignment of 47 sequences. Each motif carries 21 amino acids. Detailed localizations of all three motifs are marked on sequence alignment figure in Additional file 1. Motif M1 is on large subunit whereas M2 and M3 are localized on small subunit of GGT. As a reference, the identified motifs of *E. coli* GGT are labeled below each motif

To explore other sequence features that are conserved in GGT proteins, the alignment was subjected to MEME for finding functional motifs [39]. MEME picks up three distinct motifs: first of which is located on the large subunit whereas the second and third motifs are located on the small subunit of the GGT proteins. Each predicted motif is present in all the 47 sequences and comprises of 21 amino acids (Fig. 2). The first motif (M1) is novel since it has never been reported before and the residues composing motif have not been biochemically characterized in any GGT protein so far. This motif is highly conserved on its N-terminus (GGXXXDAAV/I) but shows higher variability in the mid region (Fig. 2). The second motif (M2) is localized towards the C-terminal of the small subunit and starts with a conserved aspargine at its N-terminus. The mid region of motif M2 has a conserved ‘LSS’ stretch in all clades of Branch B1 (*E. coli*: L461, S462, S463), whereas the same region is highly variable in all clades of branch B2 i.e. “Bact3”, “Bact4ext” and “Arch” (Additional file 3). In the *E. coli* structure (2DBX), the two serine residues of ‘LSS’ region lie at the bottom of substrate binding cavity of GGT protein and have been shown to be involved in stabilizing the intermediate γ-glutamyl moiety (carboxyl group) through hydrogen bonds [17, 29–31]. An additional small region following the ‘LSS’ triad is conserved as MSP/MTP/MCP in Bact1/Bact2/Euk clades, respectively (in all clades of branch B1) but the corresponding region is again highly variable in other GGT clades of branch 2 (Additional file 3). Thus, the second motif M2 seems to be conserved specifically in GGTs of branch B1 but quite variable in GGTs of branch B2. The third motif (M3) starts with the highly conserved threonine residue of the small subunit, also known as the N-terminal nucleophile (Fig. 2 and Additional file 3). This threonine is arguably the most important catalytic residue of GGT protein and is conserved in all GGT proteins known so far. In all the prokaryotic GGTs, this threonine along with two neighboring residues is conserved as “TTH”, where the second threonine and the following histidine have been reported to help in activation of the first threonine as a nucleophile in the GGT catalytic mechanism [17, 29–31]. The corresponding motif is found as TAH/TSH in the “Euk” clade (yeast, plants and vertebrates). Although, bacterial GGTs of clades “Bact1”, “Bact2” and “Bact3”, have this region conserved as TTH, the second threonine as well as histidine is replaced by aromatic or hydrophobic residues in all GGT sequences from extremophilic bacteria and archaeal organisms (Additional file 3), suggesting that the GGTs of “Bact4ext” and “Arch” clade may exhibit significant differences in the activation of the nucleophilic threonine, thereby affecting the kinetics of the enzyme reaction. Another small region of this motif, present as TXN in the *E. coli* GGT sequence, has been earlier shown to participate in the enzyme reaction. The threonine and the asparagine residues form hydrogen bonds with amino group of the γ-glutamyl substrate [17, 29–31]. These two residues though conserved in “Bact1” and “Euk” clades, are modified to TXS/E in “Bact2” and “Bact3” clades, whereas these are replaced by SXY/F in “Bact4ext” and “Arch” clades. Thus, once again, the GGT sequences of “Bact4ext” and “Arch” clades show replacement of charged polar residues to amino acids with hydrophobic and aromatic properties which would cause functional differences among the “Bact4ext” and “Arch” GGTs compared to GGTs of other clades.

Other than the three motifs picked up by MEME, we were able to identify another small stretch of amino acid (GXXGG), which shows high conservation in alignments (Additional file 3). The last two ‘glycine’ residues of this motif are part of the oxyanion hole and known to play significant role in stabilization of substrate intermediate complex formed during GGT enzyme reaction [17, 29–31]. It is evident from the multiple sequence alignment that the three glycine residues of this small motif are conserved in all GGT proteins but “XX” part of the motif varies in a clade specific manner. In prokaryotic GGTs (“Bact1”, “Bact2” and “Bact3”), XX is represented by SP (serine-proline), in “Bact4ext” clade this XX motif is occupied by VM (valine-methionine), whereas in archaeal GGTs (“Arch” clade) the same was replaced by CA (cysteine-alanine) and the corresponding position is occupied by AS (Alanine-Serine) in “Euk” clade. In the second GGT-like protein from both *B. subtilis and B. licheniformis*, which clusters between “Arch” and “Bact4ext” clades, this XX position is occupied by TQ (Threonine-Glutamine) (Additional file 3).

Since we repeatedly observed that many polar charged/uncharged residues in GGT change to hydrophobic residues in GGTs of Arch/Bact4ext clade, we wondered if this was a general property of the extremophilic GGTs and if the known functional residues forming the cavity of the GGT protein also showed a hydrophobic character. Therefore, we analysed the known functional residues with respect to their physiochemical features in all GGT proteins for which the structures are known in PDB database (Table 1). It was observed that out of sixteen residues, *E. coli* GGT had 10 polar and 6 nonpolar, *H. pylori* GGT had 10 polar and 6 nonpolar, *B. subtilis* GGT had 10 polar and 5 nonpolar, *B. anthracis* GGT had 10 polar and 5 nonpolar, human GGT had 11 polar and 5 nonpolar, *A. thailana* GGT had 10 polar and 6 nonpolar, *S. cerevisiae* GGT had 13 polar and 3 nonpolar, *T. acidophilum* GGT had 6 polar and 9 nonpolar, *B. haloduran* GGT had 7 polar and 8 nonpolar and *S. sulfataricus* GGT had 6 polar and 9 nonpolar residues. Thus, this analysis shows a higher ratio of nonpolar to polar residues in extremophilic and archaeal GGTs. Such differences highlight the comparatively higher hydrophobic character of the binding cavity in extremophiles and archaeal GGTs, as compared to the GGTs from prokaryotes and eukaryotes. Such differences in binding cavity of GGTs may be responsible for crucial differences in the substrate recognition/specificity and binding affinities observed in earlier studies [40, 41].

**Table 1:**
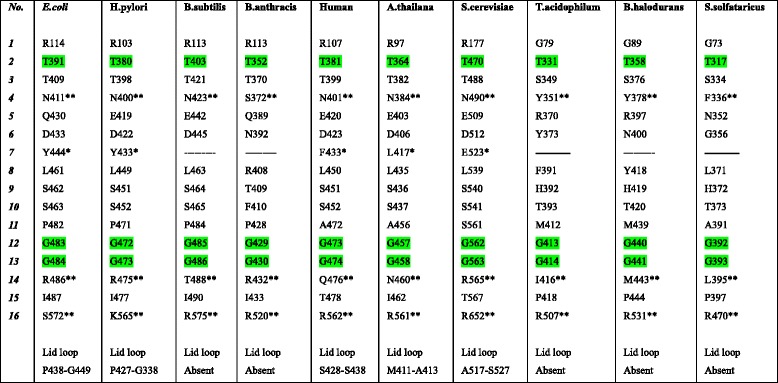
Known functional residues comparison of GGT-family: Comparative functional residues of GGT-family proteins are identified through multiple sequence alignment of GGT proteins sequences (Additional file 1)

The conserved residues throughout the family are highlighted by green color. Residues highligted with * are part of the lid loop known to be responsible for the transpeptidase activity. Residues represented by ** are proposed acceptor sites binding residues known for GGT transpeptidase activity

Overall, results of motif and sequence conservation analysis show that the residues in the neighborhood of amino acids already known to affect GGT function vary in a clade specific manner. Conservation of such amino acids within the clade but their variation in clade specific manner suggests that these residues play a significant role in imparting clade specific differences in the enzyme activity and stability of GGTs. Further experimental validation of these sites in a systematic manner should be able to delineate their role in GGT function. The identification of novel motif, M1, suggests that the large subunit of GGT may have a role in structural integrity and function of GGT proteins, which has not been addressed in any previous studies so far.

### Detection of positive selection in GGT-family

In GGT family, to explore the possibility of distinct sites in the protein being under positive selection, we used a branch-site model for sequence evolution and compared the likelihood ratio of two models M2 (null model), M2a (alternate model) and M0 (one ratio model). M0 implies a constant rate of evolution (ω = dN/dS = 0.44) for all branches (Table 2). The second hypothesis, model M2a, is used for the detection of specific-sites involved in positive selection as evident by their higher dN/dS (ω >1). In our analysis we tested M2 and M2a models hypothesis for “Bact 1”, “Bact 3”, “Bact4ext” and “Euk” clades and in all the cases we received very high dN/dS (ω_1_) ratio of 999.000 (Table 2), implying that the evolution of these clades (“Bact 1”, “Bact 3”, “Bact4ext” and “Euk”) was driven under positive selection, further suggesting the possibility of functional divergence among various clades of GGT family.

**Table 2:** Results of LRT for positive selection analysis in GGT-family members of selected clades

Foreground Branch	Model	Parameter estimated (frequency ‘f’, omega ‘ω’)					Ln L	2∆LnL for LRT Pairs M2/M2a
	Model, M0	ω_0_ =ω_1_=0.44					-23188.93	
	(ω_0_ = ω_1_)							
**Bacteria 1**		Site class	0	1	2a	2b		68.53
	Branch site	f	0.20	0.23	0.26	0.30	-22809.80	
	Model, M2	ω_0_	0.25	1.00	0.25	1.00		
	(0<ω<1)	ω_1_	0.25	1.00	1.00	1.00		
		Site class	0	1	2a	2b		
	Branch site	f	0.28	0.32	0.19	0.21	-22775.54	
	Model, M2a	ω_0_	0.25	1.00	0.25	1.000		
	(0<ω<1)	ω_1_	0.25	1.00	999.00	999.00		
	Model, M0	ω_0_ =ω_1_=0.44					-23188.93	
	(ω_0_ = ω_1_)							
**Bacteria 3**		Site class	0	1	2a	2b		69.99
	Branch site	f	0.21	0.25	0.25	0.29	-22805.67	
	Model, M2`	ω_0_	0.25	1.00	0.25	1.00		
	(0<ω<1)	ω_1_	0.25	1.00	1.00	1.00		
		Site class	0	1	2a	2b		
	Branch site	f	0.20	0.24	0.26	0.30	-22770.68	
	Model, M2a	ω_0_	0.25	1.00	0.25	1.00		
	(0<ω<1)	ω_1_	0.25	1.00	999.00	999.00		
	Model, M0	ω_0_ =ω_1_=0.44					-23188.93	
	(ω_0_ = ω_1_)							
**Bact4ext**		Site class	0	1	2a	2b		69.90
	Branch site	f	0.21362	0.24819	0.24895	0.28924	-22805.67	
	Model, M2	ω_0_	0.24612	1.00000	0.24612	1.00000		
	(0<ω<1)	ω_1_	0.24612	1.00000	1.00000	1.00000		
		Site class	0	1	2a	2b		
	Branch site	f	0.21	0.24	0.25	0.29	-22770.72	
	Model, M2a	ω_0_	0.25	1.00	0.25	1.00		
	(0<ω<1)	ω_1_	0.25	1.00	999.00	999.00		
	Model, M0	ω_0_ =ω_1_=0.44					-23188.93	
	(ω_0_ = ω_1_)							
**Eukaryotes**		Site class	0	1	2a	2b		111.79
	Branch site	f	0.16	0.19	0.30	0.35	-22807.60	
	Model, M2	ω_0_	0.25	1.00	0.25	1.00		
	(0<ω<1)	ω_1_	0.25	1.00	1.00	1.00		
		Site class	0	1	2a	2b		
	Branch site	f	0.09	0.10	0.37	0.43	-22751.71	
	Model, M2a	ω_0_	0.26	1.00	0.26	1.00		
	(0<ω<1)	ω_1_	0.26	1.00	999.00	999.00		

Positive selection analyses (M0, M2 and M2a) are performed by using ‘Codeml’ implemented in PAML. LnL: log likelihood. LRT: likelihood ratio test. 2∆lnL: twice the log-likelihood difference of two compared models. Model M2 and M2a are tested for null hypothesis and alternate hypothesis respectively. The significant tests are performed at level of significance cut-off value of 1 % using BEB statically methods. Null hypothesis was tested by fixing omega equal to 1. 2∆LnL = 2(LnL^M2^- LnL^M2a^)

### Functional divergence in GGT-family

Since the ‘Codeml’ [42] results suggested a possibility of functional divergence among the GGT family members, we next attempted to identify putative sites which might be responsible for type 1 (FDI) and type 2 (FDII) functional divergence in GGT proteins. In the current study, the divergence analysis was performed on six pairs of GGT clades on the phylogenetic tree and significant values of divergence coefficient for FDI and FDII analyses (θI and θII >0) for all pairs were obtained (Table 3 and Additional file 4). Here, we discuss two cases (Bacteria 1 vs. Bacteria 4-Extremophiles and Bacteria 1 vs. eukaryotes) in more detail because they appear to be more important from the protein engineering and drug design point of view.

**Table 3:** Divergence analysis for GGT subfamilies: Divergence type I and type II analyses of two clusters comparisons are shown here in details

Bacteria 1/Bacteria 4-Extremophile						Bacteria 1/Eukaryote					
**FD I**			**FD II**			**FD I**			**FD II**		
θ I = 0.49 ± 0.08			θ II =0.31 ± 0.09			θ I = 0.58 ± 0.12			θ II = 0.31 ± 0.08		
P = 0.75			R = 9.50			P = 0.70			R = 10.35		
**Site**	***E c***	***B h***	**Site**	***E c***	***B h***	**Site**	***E c***	***Hm***	**Site**	***E c***	***Hm***
**G 1**	**G 1**	**G 1**	**G 1**	**G 1**	**G 1**	**G 1**	**G 1**	**G 1**	**G 1**	**G 1**	**G 1**
415	P291	P268	215	R114	S88	544	V396	V386	540	T392	A382
463	A335	V309	540	T392	V359	546	D398	A388	631	S481	A471
465	R337	G311	541	H393	Y360	629	T479	V367	638	I488	T478
474	F346	D320	555	T407	I374	632	P482	A472			
478	D350	S319	559	N411	Y378	641	V491	T481			
535	E387	P354	611	S462	I422	725	I556	I547			
576	N428	Q395	631	S481	V438	758	E563	G553			
583	S435	S402	632	P482	M439						
602	A453	A410	637	I487	Q444						
			638	I488	P445						
**G 2**	**G 2**	**G 2**	**G 2**	**G 2**	**G 2**	**G 2**	**G 2**	**G 2**	**G 2**	**G 2**	**G 2**
264	Y161	Y133	184	A84	T59	163	I65	A57	184	A84	G76
275	V170	L142	187	H87	T64	287	I182	P169	187	H87	N79
276	Q171	A143	190	A90	N66	342	L231	Y217	188	P88	A80
287	I182	P154	196	G95	D71	383	A269	N255	190	A90	S82
349	G238	N215	213	D112	N86	409	Y285	V272	191	G91	M83
351	D240	E217	216	E115	G89	491	K361	A352	197	G97	L89
398	R283	R260	338	L227	A205	647	N497	Y487	213	D112	N105
409	Y285	Y262	389	R275	V252	687	D532	A522	249	G147	A133
432	E308	K284	543	S395	A362	719	G551	A542	261	L158	H145
440	G316	D292	579	M431	G398				264	Y161	H148
486	N356	S330	641	S465	H419				266	T163	R150
487	K357	D331	665	H515	Q472				278	A173	S160
489	Y359	Y333							316	K206	C192
545	V397	A364							362	A248	T234
550	N402	N369							389	R275	I261
624	K474	Q431							395	G280	I267
649	I499	I456							398	R283	G270
675	D521	K477							408	G284	D271
687	D532	D488							414	M290	P277
759	L564	L524							418	P292	A279
									416	S294	L281
									423	H299	V285
									432	E308	K294
									464	D336	K326
									487	K357	E348
									585	K437	P427
									608	R459	Q448
									643	Q493	L483
									650	D500	W490
									675	D521	N511
									761	G566	A556

θI and θII >0 are observed for type I and type II analysis respectively in each case. Corresponding residues are marked on the two representative proteins of the compared clusters with respect to predicted divergence sites by FDI and FDII methods. Divergence sites predicted through the analysis are divided into two groups, G1 and G2, where G1 represents the sites that are close to the known functional residues forming the substrate binding cavity and G2 represents all other sites.  *Ec*: gamma glutamyl transpeptidase of *Escherichia coli. Bh*: Gamma glutamyl transpeptidase of *Bacillus halodurans. Hm*: gamma glutamyl transpeptidase of human

### a) Bacteria 1 (B1) (*E. coli*) vs. Bacteria 4-Extremophile (B2) (*B. halodurans*)

**Fig. 3:**
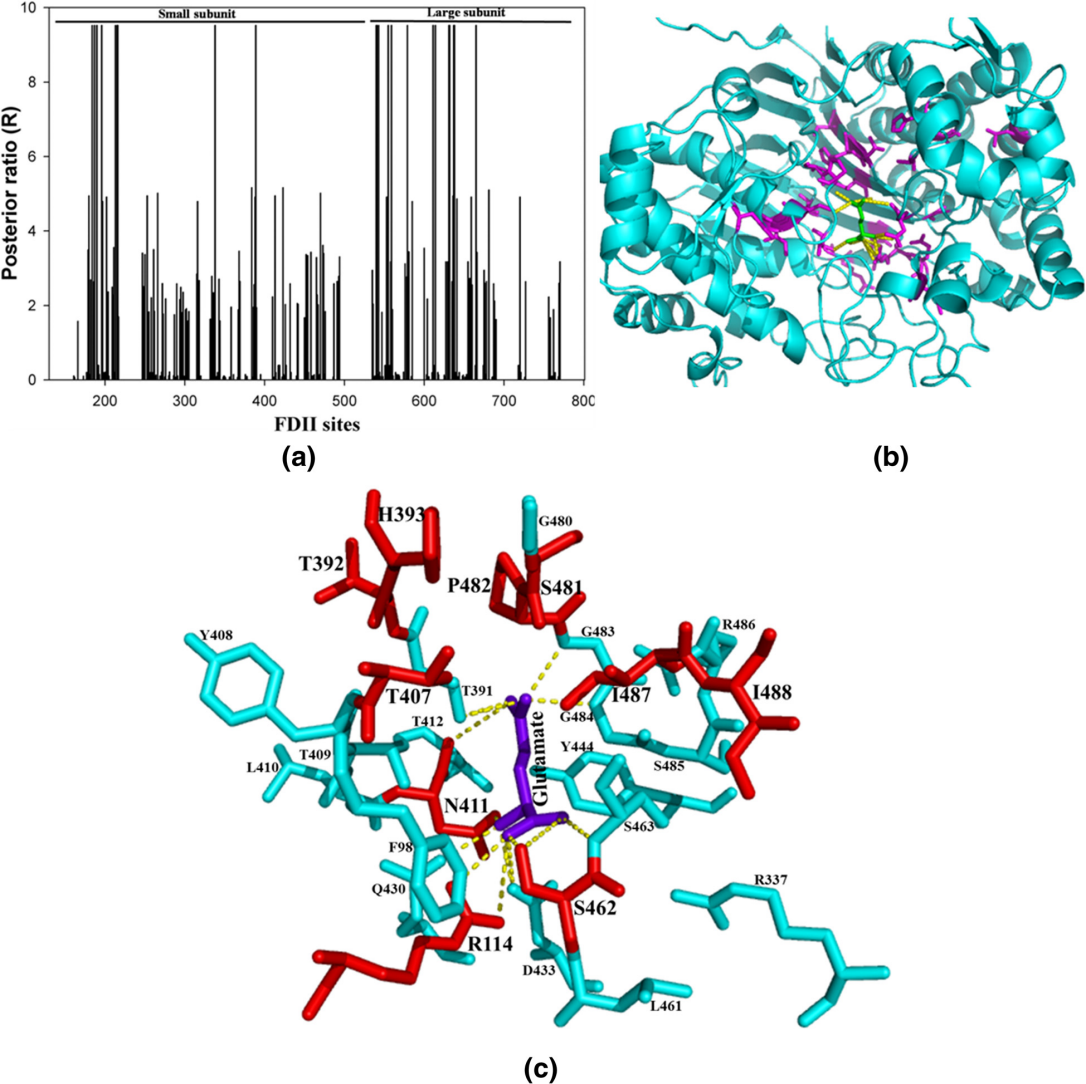
Type 2 functional divergence analysis for Bacteria 1 vs. Bacteria 4 extremophile. **(a)** Twenty two amino acids type 2 divergent sites are observed at posterior ratio (R) 9.5. The putative divergent sites are marked as a highest peak in figure. Nine sites are observed on large subunit and thirteen are on the small subunit. **(b)** The type 2 divergent sites predicted by DIVERGE 3.0 amino acids sites are mapped on GGT structure (cyan color) of *E. coli* (2DBX). The identified FDII sites are shown in stick (magenta) whereas the inbuilt glutamate substrate is shown in green stick. **(c)** The FDII sites identified in Bacteria 1 vs. Bacteria 4 extremophile cluster comparison are mapped on glutamate (violet) binding cavity (cyan color) of *E. coli* GGT within the 6 Å radius. Ten divergent sites (red color) out of twenty four type 2 predicted divergent sites are observed in binding cavity region of *E. coli* GGT

Here, functional divergence analysis was used to predict sites which might be responsible for key functional differences between the members of two clades “Bact 1” and “Bact4ext”. The functional divergence type 1 analysis of “Bact 1” vs. “Bact4ext” clusters revealed 29 distinct divergence sites at threshold cut off of posterior probability (p) > 0.75 (θI = 0.49 ± 0.08) (Table 3). For the same clusters, FDII analysis is highly significant with a value of θII = 0.31 ± 0.09 and revealed 22 putative divergent sites at threshold posterior ratio (R) of 9.5. To analyse the possible role of these amino acid changes between the two clusters, the sites identified by FDII were mapped on the structure of *E. coli* GGT (Fig. 3b). It was observed that nine of these putative sites are on large subunit and thirteen are on the small subunit (Fig. 3a). We observed that ten of these predicted sites are part of loops, six are part of α-helices and the remaining six sites lie on the β-strands (Fig. 3b). Further, it appears that majority of the putative sites lie near the substrate binding cavity of the GGT-protein. We therefore used the *E. coli* GGT structure (2DBX) to generate a potential substrate binding cavity by creating a 6 Å radius around the bound glutamate (Fig. 3c). Twenty four amino acid residues lie within this radius, where ten of these have been predicted to be type 2 divergent sites (coloured red in Fig. 3c). This indicates that there is substantial contribution of divergent sites in defining the binding cavity of the GGT enzymes of these two clusters. Such sites could potentially be responsible for differences shown by the enzymes of these two clades in terms of transpeptidase activity, which could be further confirmed by site directed mutagenesis experiments in lab. Interestingly, the putative divergent sites (631, 632: S481/V442, P482/M443; *E. coli/ B. halodurans*) predicted by FDII are localized on the motif GXXGG described earlier in this work. Here, serine and proline of *E. coli* protein are substituted by the more hydrophobic residues like valine and methionine in extremophile (Bact4ext) clade. Other than these, few more sites are part of motifs described earlier, like A72 and L83 (from *E. coli*) are part of the motif M1, V469 is part of motif M2 whereas D398 and A405 are present on motif M3.

### b) Bacteria 1 (*E. coli*) vs. eukaryotic (human) GGTs

**Fig. 4:**
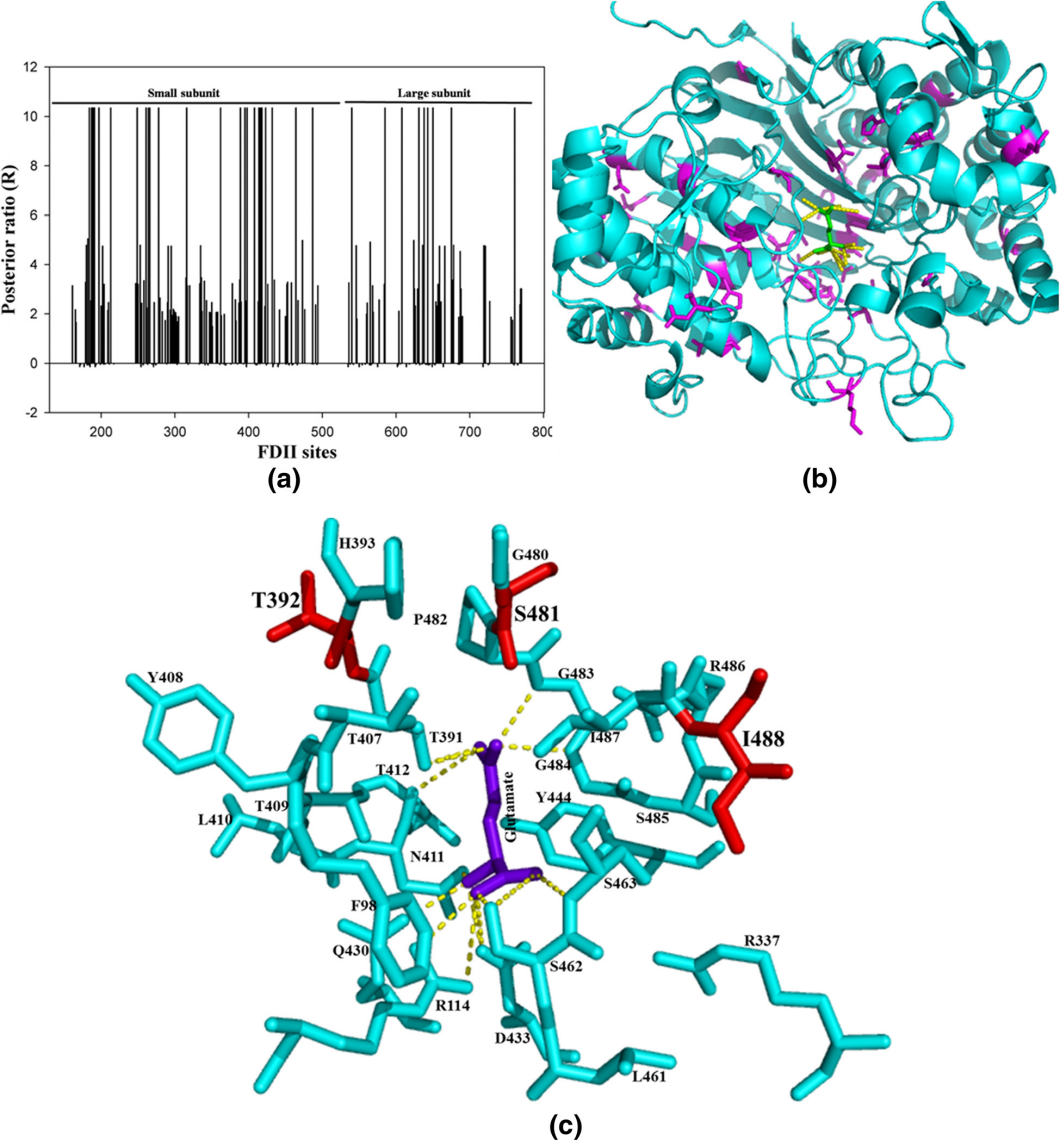
Type 2 functional divergence in Bacteria 1 vs. Eukaryote. **(a)** Thirty four divergent sites at posterior ratio (R) 10.35 have been observed from FDII analysis of prokaryote I vs. eukaryote clusters. The putative divergent sites are marked as a highest peak in figure. The twenty five sites are observed on large subunit and nine are on the small subunit. **(b)** The type 2 amino acids sites are identified by DIVERGE 3.0 mapped on GGT structure (cyan color) of *E. coli* (2DBX). The identified FDII sites are shown in stick (magenta) whereas the inbuilt glutamate substrate is shown in green stick. **(c)** The FDII sites identified in Bacteria 1 vs. eukaryotes cluster comparisons are mapped on glutamate (violet) binding cavity (cyan color) of *E. coli* GGT within the 6 Å radius. The putative 3 divergent sites (red color) of functional divergence type 2 analyses are observed in binding cavity region of *E. coli* GGT

Earlier studies on physiological role of GGT have highlighted their medical importance in both the mammalian as well as microbial GGTs. Inhibiting human GGTs has been shown to make chemotherapy more effective while inhibiting microbial GGTs has been shown to impede their pathogenicity. Thus, the comparison between bacteria 1 (mostly pathogenic bacteria) and eukaryotes clusters appears to be of great interest in being able to identify potential sites that differ in the two clades. Such sites could potentially be helpful in designing novel inhibitors against specific GGTs. The FDI analysis for these clusters revealed significant value of θI = 0.58 ± 0.12 > 0 and at a threshold cut off of p >0.7 (P < 0.01, Fisher transformation test), 16 sites are observed, which are conserved in one cluster but are variable in the other cluster (Table 3). The signal for type 2 functional divergence is strong with a significant value of θII = 0.31 ± 0.08 and at threshold posterior ratio (R) of 10.35, 34 divergent sites could be identified (Table 3, Fig. 4a). To gain more insight into the possible function of these predicted FDII sites, we mapped these sites on the structure of *E. coli* GGT (Fig. 4b). Ten of these sites are lie on α-helices, five are on β-strands and the other nineteen are part of the loop regions (Fig. 4b). It was observed that three of these type 2 sites lie within the binding cavity (6 Å cavity around the bound glutamate molecule) of *E. coli* GGT, thus actively contributing to the changes in the local environment of this cavity (Fig. 4c). Interestingly, the divergent site (631, S481/A471; *E. coli*/human) are part of the oxyanion hole containing motif GXXGG, thus suggesting that the significant differences observed in the rate of reaction between the proteins of these two clusters may be result of these changes. The FDII predicted sites A84, H87 and P88 (*E.coli* GGT structure) have been observed on motif M1, R459 is part of motif M2 and T392 lies on motif M3, again suggesting that these sites significantly contribute to protein function and structure. Interestingly, in this case a large numbers of predicted divergent sites are part of the large subunit (twenty five sites), which implies that the large subunit plays an important role in functional divergence between the GGTs of these two clusters (Fig. 4a & 4c). The large subunit has largely been neglected in all the previous studies as the catalytic activity of the GGT molecule has been thought to come solely from the small subunit. Our results however, strongly contend the need to characterize the role of large subunit in generating functional diversity in the GGT proteins. The amino acid differences observed in the binding cavity of this protein may be helpful in designing specific inhibitors against the mammalian and microbial GGTs.

### c) Functional divergence in other GGT subfamilies

Similar FDI and FDII analysis was also carried out for four other pairs of clades, e.g. “Bact1” vs. “Arch”, “Bact1” vs. “Bact3”, “Bact4ext” vs. “Arch” and “Bact3” vs. “Arch”. For all the above mentioned subsets, the values of θI and θII were greater than 0, thus suggesting that strong functional divergence signals could be picked up between these clusters (Additional file 4). The sites predicted from such analysis of these subclusters of GGT-family are shown in Additional file 4. Organisms in bacteria 1 clade are mostly pathogenic and their GGT proteins contain the lid-loop structure which is missing from the GGTs of the Bacteria 3 clade. The detailed analysis of these sites would help in delineating the amino acids that are responsible for differences in biochemical features and structural stability in GGTs of these clades. Also, comparison of the archaeal sequences with various bacterial clades could possibly highlight the amino acid residues which may impart extra stability to such enzymes, as GGTs characterized till date from various archaeal organisms show high thermostability.

## Conclusion

Our phylogenetic analysis of GGT proteins from different organisms divides the GGTs into various clades and offers several interesting insights into the evolution and relatedness of these GGTs. Sequence conservation studies and motif detection show that most of the highly conserved residues have been characterized biochemically in various previous studies and are already known to be functionally important for the enzyme activity of this protein. However, the present study focuses on the residues that are highly specific to each GGT subfamily and underlines their importance in imparting unique functional properties to the GGT proteins of each clade.

The present study highlights the clade specific variation in the GXXGG motif, where SP (XX) of bacterial GGTs is substituted by VM, CA, AS in extremophilic bacteria, archaea, and eukaryotes respectively, which could explain the differences in rates of enzyme reaction in GGTs of these clades as this motif is known to be involved in GGT-substrate complex intermediate formation and the rate of final product release [17, 30]. We have been able to identify another motif on the large subunit of the GGT protein, which has never been reported before and none of the amino acids that are part of this motif have been biochemically characterized earlier. Detailed studies targeting the role of these amino acids could finally reveal the possible role of large subunit in the overall function and stability of the GGT protein. The fact that large subunit has an important role to play in GGT function and structure is also underlined by the large number of type 2 divergence sites picked up large subunit of GGT protein while comparing the eukaryotic and bacteria 1 GGT clades.

Our analysis also shows that many sites predicted to be contributing to type 2 functional divergence are quite often found lining the substrate binding cavity and are close to the highly conserved known functional residues. This implies that they may be affecting the biochemical environment of the binding cavity and influencing the catalytic residues, thereby contributing to the functional differences among GGT-like proteins of various clades. Similarly, the putative divergent sites identified on the backbone of these proteins may be contributing to differences in structural stability. We propose that studying functional divergence between various clades could possibly highlight residues that are part of the GGT acceptor site, the complete identification of which has eluded researchers so far. Further biochemical characterization of the putative divergent sites identified by our study could help in delineating the role of these amino acids in imparting thermal/pH stability and those responsible for differences in enzyme kinetics and substrate specificities. Because many of the putative divergent sites are found to line the substrate binding cavity of the GGT protein, we expect that this knowledge could also be useful in design of inhibitors that are clade/species specific. Therefore, identifying functional divergence hotspots in the proteins could possibly augment the structure based drug-design approaches in general.

## Methods

### GGT protein sequences and phylogenetic tree analysis

The complete cDNA and protein sequences of GGT-family were retrieved from NCBI database. Aminoacid sequences of GGTs that have been biochemically characterized earlier (*E.coli* GGT; AAA23869, *H.pylori* GGT; AAG34111, *B.subtilis* GGT; BAM52495, B.anthracis GGT; BAA03126, *B.halodurans* GGT; NP_241733, *T.acidophilum* GGT; CAC12123, and Human GGT; NP_038347, *S.cerevisiae* GGT; AAB67344) were used to find similar sequences using BLASTp. Since no GGT has been biochemically characterized in detail from plants and archaea, the GGT from model organisms *Arabidopsis thaliana* (AtGGT; CAA89206) and *Sulfolobus solfataricus* (SsGGT; NP_344523) were used as seed sequences respectively for plant and archaeal kingdoms, and the top five related sequences obtained by BLASTp were included in this comparative study. All sequences for which structural information is available have also been included. Thus, in the present study, GGT representative sequences from three domains: prokaryotes, eukaryotes and archaea are included. Two bacterial organisms (*Bacillus subtilis* and *Bacillus licheniformis*) have two GGT like genes, which have both been also included with the intention of comparison.

A PSI-BLAST search against the NR database, to find sequences distantly related to the seed sequences, results in more than 20,000 sequences. The Pfam database also has 21767 GGT-like proteins (as on 10/07/2015), but this dataset is highly redundant. When CD-HIT was used to reduce redundancy at a cutoff of 100 %, the number of sequences reduces from 21767 to 8117 [43]. At 50 % identity cut-off, 760 sequences are left, many of which are partial and short, removing which leads to a dataset of 487 sequences. This subset achieved is however too diverse and the resulting tree does not give any stable separation of sequences into clades. Such a subset defeats the purpose of the proposed study since it collects only the most diverse members and fails to highlight any significant similarities between the members of any clade. The main objective of the sequence selection had to be inclusion of sequences that have already been characterized biochemically, and to select sequences close to these seed sequences so that we can expect them to be functionally similar to the seed sequences. This then helps to amplify the signal of residues conserved in one clade versus the other clade.

The final dataset comprises a total of 47 GGT protein sequences, which were aligned using different algorithms like T-coffee, ClustalW, and MUSCLE [44–46]. An alignment score of 96 (by T-coffee), showing that the quality of alignment is good, was achieved after some manual editing in Bioedit [47]. Additionally, multiple sequence alignment was also performed by sequence alignment packages inbuilt in MEGA 5.10 [48]. The conserved motifs among these protein sequences were identified by MEME server using default parameters [39]. Based on the manual observations and highest alignment score, the multiple sequence alignment done by MUSCLE was taken as the final alignment. The average length of GGT sequences included in this analysis was 565 amino acids.

The phylogenetic trees of these 47 GGT proteins sequences were constructed by maximum likelihood (ML) and neighbor joining (NJ) methods based on gamma corrected Jones-Taylor-Thornton (JTT) with boot strapping of 1000 steps. The resulting trees resolved the GGT proteins into two main branches, B1 and B2, with each branch being further divided into three distinct clades. All the six main clades achieved 99 % boot strap values in the ML phylogenetic tree. Internal nodes of these clades have boot-strap values > 55 % in the ML phylogenetic-tree suggesting that the overall quality and strength of the constructed tree is good. ProtTest was used to choose the best-fitting model to build the phylogenetic tree [49].

### Detection of positive selection in gamma glutamyltranspeptidase subfamily clusters

The conservation of few amino acids in a clade wise manner indicates that the proteins in various clades may have adapted to different functions. Whether these clades are under selective pressure for functional divergence can be predicted by observing changes in the evolutionary rates and to test this hypothesis we used ‘Codeml’ program [42]. In this method, a certain clade (prokaryotes, eukaryotes, extremophiles, and archaea) was selected as foreground set for which a positive selection model was applied while the null model was applied for the other clades of tree (considered as background). The data used for detection of positive selection in gamma-glutamyl transpeptidase family was generated by selecting a subset of 23 DNA sequences from the earlier data set of 47 sequences. The selected subset was based on the priority of higher diversifications roots observed in phylogenetic tree. Aligned DNA sequences were back-translated to MEGA 5.10 to achieve a codon alignment. Phylogenetic tree for this subset was constructed by Phyml [50]. The codon alignment and phylogenetic tree were used as input data to run Codeml analysis implemented in PALM 4.6 program package [51]. The Codeml program predict the measurement of the relative rate of nonsynonymous (dN) to synonymous substitution (dS), or ω = dN/dS, using Nei–Gojobori method [52]. The branches likely undergoing positive selection were predicted by using branch models implemented in Codeml program of PALM package. In Codeml analysis, we assumed that the branches of our interest have different dN/dS ratio as compared to rest of the three branches. In this study we compared three models namely M0, can be called as one ratio model where all branches have equal rate (dN/dS = constant value), M2 (null model) was used to detect null hypothesis and M2a (alternate model) model used for detecting positive selection or acceleration in the post duplication branch forming the novel subfamilies of GGT and third model [53–56]. Both M2 and M2a were compared on the basis of their LRT values.

### Divergence analysis to detect sites contributing to functional changes in GGT subfamilies

The sites contributing to the type 1 functional divergence (FDI) are the ones that are highly conserved in one cluster but are variable in the other cluster, thus reflecting changes in evolutionary rates between the two clusters at these sites. The type 2 functional divergence (FDII) analysis predicts sites which although conserved in both clusters but are quite distinct in their biochemical features, thereby accounting for functional shifts between the two clusters. DIVERGE 3.0 was used to predict the functional divergence between the different clades of the GGT-family [57]. In this study we analyzed two types of functional divergence, known as type 1 and type 2 [58–62]. Multiple sequence alignment of GGT sequences are subjected to DIVERGE 3.0 and a rooted NJ tree was generated using Poisson correction distance measure. Cluster size of each subfamily was fixed to > 4 sequences. The effective numbers of functionally related divergent sites are defined as minimum number of sites, such that when they are completely removed, the coefficient of functional divergence (θ) for the rest of sites approaches to zero. The site specific posterior probability cut off values of type 1 and type 2 functional divergence sites were obtained by conventional methods. Here we ranked amino acid sites according to their posterior probability and the corresponding coefficient of functional divergence (θ) is calculated. Then the amino acid site exhibiting highest posterior probability was completely removed from MSA file using Bioedit and then the coefficient of functional divergence (θ^*^) is recalculated. The same steps are repeated until the condition of θ^*^ approaching to zero is satisfied or θ^*^ satisfy the condition of θ^*^ < se^*^ where se^*^ is the standard error of the θ^*^ used to control the long tail problem. When the θ^*^ < se^*^ condition is achieved, the calculation coefficient of functional divergence (θ^*^) step was terminated. The removed specific amino acids sites were counted as effective functional divergence sites between the two subfamilies of GGT family. In this study, the value of divergence coefficient (θ) achieved is significantly larger than zero, indicating good functional divergence signal between the clusters. Further, we analysed the divergence sites by positioning these sites on the 3D structures of GGT proteins of *E. coli* (2DBX) and human (4GDX). Pymol molecular viewer was used for 3D visualization of proteins [63].

## Reviewers comment

### Reviewer 1, first report (Dr Andrei Osterman, Burnham Institute, United States of America)

The article provides a rather detailed analysis of evolutionary divergence and conservation of certain structure-functional features within a very interesting and uniquely ubiquitous gamma glutamyl transpeptidase enzyme family. Despite the depth of the analysis, eloquent use of available tools and well-organized presentation of the main findings, the actual impact of the manuscript appears questionable. Even the selection of 47 representative species (given thousands available genomes) is not clearly justified, and, to make things worse, may introduce some biases at the level of conclusions. E.g., the most striking example of a potential bias is the statement (page 5) that “The“Bact1” clade, exclusively composed of bacterial organisms known to be pathogenic for human…” A closer look at the list shows that the entire branch is simply composed of Proteobacteria, and all selected representatives just happen to be related to known pathogens (while myriads of nonpathogenic Proteobacteria were simply not included in the analysis!). Moreover, a number of potentially interesting and important aspects are just mentioned in passing while many other rather technical and mundane observations are described in detail. For example “valuable functional or physiochemical differences” or “biotechnological and biomedical applications” of GGT enzymes are mentioned several times, but no details and no clear functional associations with the observed structural variations (emerging from this analysis) are conveyed. As a result, the overall impression from this paper is that the authors laid out a nice framework for the actual predictive analysis (to be followed by experimental testing) but stopped short of any real breakthroughs in structure-functional or mechanistic understanding of the family or its particular representatives. Thus, while they duly emphasize the utility of comparative analysis for supporting the development of specific inhibitors, the paper hardly provides any tangible guidelines for such development (beyond pointing to clade-specific variations in the vicinity of universally conserved residues). To make it simple, the current version of a paper comes thru as an introduction or a setup for a story, but THE STORY itself remains untold. This overall impression holds despite a few interesting observations on evolution (e.g. on large similarity between Bact1 and Euk branches as compared to many other Bacteria) or patterns of sequence conservation/ variation as they do not seem to lead to a new level of understanding. Moreover, all methodology used in the paper is rather established, and it is unlikely that yet another illustration of its utility on the example of a particular family (no matter how important) would be of sufficiently broad interest.

**Quality of written English:** Acceptable.

**Author’s response:***We thank the reviewer for his appreciation of the use of tools and in-depth analysis.*

*As far as the sequence selection is concerned, it seems that we have not been able to clearly communicate our methodology and intent of sequence selection since all the three reviewers have similar queries. Please let us clarify again that we have indeed grappled with the issue of including “diversity” vs. “selecting closely related sequences” for quite some time during the initial part of the studies. This is reflected in the mistake in the “Methods section” where we mention that sequences were selected through PSI-BLAST. Indeed, we had tried PSI-BLAST to see what variety of sequences we would get from the databases and as mentioned by another reviewer, we do get more than 20,000 sequences. The Pfam database also has 21767 GGT-like proteins (as on 10/07/2015) but this dataset is highly redundant. When we try to narrow them down to a smaller set through CD-hit like approaches, a cutoff of 100 % reduces the numbers from 21767 to 8117 sequences. At 50 % identity cut-off, 760 sequences are left, many of which are partial and short, removing which leads to a dataset of 487 sequences. This subset achieved is however too diverse and the resulting tree does not give any stable separation of sequences into clades. Such a subset defeats the purpose of the study since it collects only the most diverse members and fails to highlight any significant similarities between the members of any clade. This kind of alignment gives us information about residues that are conserved across all GGTs, like the catalytic center threonine and two glycines. This information is available for last many years and does not add anything now to our understanding of functional variability among different GGTs. The main objective of the sequence selection thus had to be inclusion of sequences that have already been characterized biochemically, and to select sequences close to these seed sequences so that we can expect them to be functionally similar to the seed sequences. This then helps to amplify the signal of residues conserved in one clade versus the other clade. All sequences for which structural information is available have also been included. Since no GGT-like protein has been structurally or biochemically characterized yet from archaea and plants, so in these cases we took the GGT protein from the model organism of each domain (A. thaliana (plant) and S. solfataricus (Archaea)) as the seed sequence. The closest homologs of these GGT sequences were used to generate representation from the three domains of life. We have now edited the Methods section to remove the comment about PSI-BLAST usage and we hope that this clarifies our methodology of selection of GGT sequences.*

*To address reviewer’s comment about “entire branch is simply composed of Proteobacteria, and all selected representatives just happen to be related to known pathogens (while myriads of nonpathogenic Proteobacteria were simply not included in the analysis!)”, we would like to draw attention to the fact that at least two members of this clade; “Eubacteriaceae bacterium” and “Peptoniphilus indolicus”, belong to Firmicutes division, so the “Bact 1” clade is not simply “Proteobacteria”. Also on reviewer’s suggestion, we chose sequences from five nonpathogenic proteobacteria and recreated the tree by using Maximum likelihood (ML) method of MEGA. In the new tree, the sequences from these “nonpathogenic proteobacteria” form a separate clade on the tree close to Bacteria 2 clade but far from the “Bact 1” clade whereas all other clades of the tree remain unchanged (Additional file**5**: Figure S3). This concurs with our earlier observation.*

*We can understand the reviewer’s point of view when he mentions that the “actual story remains untold”. However, the present analysis was only meant to investigate if the approach of comparing GGT proteins (which exhibit functional variability) through “functional divergence analysis” could provide us any leads into what residues would be responsible for this diversity of enzyme activity. In the study, we have enough circumstantial evidence to suggest that this approach may indeed serve its purpose. This has been corroborated by reviewer himself when he suggests that the paper does “point to clade-specific variations in the vicinity of universally conserved residues”. In our opinion, “the actual predictive analysis (to be followed by experimental testing)”, is too detailed and vast for it to be included in this one paper. It will require analysis of the possible influence of every single putative residue predicted through functional divergence between any two given clades with respect to the hundreds of mutational studies reported on the GGT proteins of these clades. It will also entail exploring the possible role of these amino acids in designing novel specific inhibitors against members of the either clade. We are already halfway through such analysis for the Bact1 vs Euk clades and hope to report some interesting findings soon. We also plan to test these mutations in the wet-lab to understand the influence of each amino acid on substrate specificity ad enzyme kinetics. Until then, we had only wanted to share our excitement over the fact that such an approach could possibly pick up residues responsible for functional differences and also lead to development of more specific inhibitors.*

### Reviewer 1, second report (Dr Andrei Osterman, Burnham Institute, United States of America)

The article provides a detailed analysis of evolutionary divergence and conservation of structure-functional features within a very interesting and uniquely ubiquitous gamma glutamyl transpeptidase (GGT) enzyme family. For this analysis, authors have selected a rather limited subset of GGT family to enable the analysis of variations within relatively compact phylogenetic groups with functionally and/or structurally characterized representatives. As a result, they built a framework for elucidation of evolutionary relationships and potentially important structure-functional variations within this family, a subject of further computational and experimental analysis. Thus, mapping of clade-specific variations in the vicinity of universally conserved residues suggests the principal possibility and provides a support for development of specific inhibitors. Among interesting evolutionary observations is a larger sequence similarity of a particular Bacterial branch of GGT family with the Eukaryotic branch as compared to its similarity with other Bacterial branches.

**Quality of written English:** Acceptable

**Author’s response:***We thank the reviewer for his positive reception.*

### Reviewer 2, first report (Christine Orengo, University College London, United Kingdom)

This manuscript presents the results of a phylogenetic analysis of the glutamyl transpeptidases (GGTs), together with other studies to detect conserved sequence motifs which were interpreted in a structural context. These analyses allowed the authors to detect several motifs comprising some residues with known biochemical characterization. Studies of sites under positive selection are also performed. Some residues identified in the studies locate to the large subunit suggesting a functional role for this subunit which has not been previously appreciated and with important considerations for drug design. The work is comprehensive, the manuscript very clearly written and the figures are all useful and well presented. The authors present a good case for the importance of gamma glutamyl transpeptidases (GGTs) and the potential value of inhibitors specific to subfamilies of these enzymes. Their choice of methods such as Codeml for maximum likelihood analysis is good and the efforts that are made to interpret their results in the context of protein structure and mechanism of action are commendable. This is an important family of proteins and the studies provide important insights into active site differences that could have potential impacts on function.

I have one major criticism of this work and several minor criticisms.

**Author’s response:***We thank the reviewer for her appreciation of the methodology and analysis.*

The major criticism of this work is that although the authors say in Methods that they use PSI-BLAST to identify GGTs, which in my hands finds many thousands of significant matches in GenBank to their seed sequences, all their analyses are restricted to just the 47 proteins that have structures in the PDB. I'm concerned that these 47 proteins are not representative of the known phylogenetic diversity of these enzymes and that their results may differ from the results of a broader analysis. I appreciate that this work largely focuses on a structure-based interpretation of the results but this does not really justify restricting the whole study, including phylogenetic analysis, to only those proteins with an experimentally determined structure when so many more sequences are available.

**Author’s response:***We are sorry about the inclusion of the comment about usage of PSI-BLAST in collecting sequences. It was indeed used in initial stages to gather all the distant variants of GGT-like proteins but it is definitely not required for collecting the subset that is used in the present study. It had been inadvertently left in the methods section, which is now edited to correct this. The selection of present subset of GGT-sequences is discussed in detail in response to the query by reviewer 1. We do hope that it addresses the query of reviewer 2 also.*

**Minor criticisms are:**

1. On page six, for example, horizontal gene transfer is suggested as being important but surely this would tend to compromise the validity of phylogenetic analysis. What evidence is there for the presence of GGT genes on plasmids etc. and how common is this?

**Author’s response:***We only meant to say that the distribution of sequences on the tree shows some interesting evolutionary paths taken by GGT-like proteins. In the case of two GGT-like proteins found in B.licheniformis and B.subtilis, horizontal gene transfer has been suggested as one of the possibilities. Truly, we havn’t really investigated the evidence for or against this hypothesis as it was not the main focus of the manuscript, although we are definitely interested in examining the role of HGT in GGT evolution in future works. At this point, we think even though this hypothesis only lends a possibility and has not been proved, it does not compromise anything on the main aim of the paper, so this comment could possibly stay. If the reviewers disagree, we are willing to edit the manuscript to remove the comment on HGT.*

2. On page 8 the authors show a higher ratio of non-polar to polar residues in extremophilic and archaeal GGTs which is interesting but I'm not sure why they don't calculate the statistical significance of this result. I would suggest that Fisher's exact test is appropriate for this small set of categorical data and in my hands their results are significant at P < 0.01.

**Author’s response:***On page 8 of the manuscript we have reported higher ratios of non polar to polar amino acids in the binding cavity of extremophilic and archaeal GGTs as compared to GGTs from eukaryotes and bacteria. In our opinion, although this observation gives us a hint that differences in the cavities of different GGTs may be related to hydrophobicity, it did not appear to do so in any statistically significant manner, thus no such test was reported earlier. After reviewer’s suggestion, we tried the Fisher’s test, where our results are significant at P < 0.01 in some cases but they are not significant at the same level in other cases. Therefore no point is being made in the manuscript for the same.*

Furthermore, the authors refer to studies in the literature reporting differences in substrate specificities and binding affinities but make no comment on the different substrates involved and whether changes in the affinities correlate with the changes in the nature of the binding site. It would be helpful if they could comment on this.

**Author’s response:***The vast literature on biochemical characterization of different GGTs generally focuses on affinity of different substrates (like glutamate, glutathione and inhibitors like acivicin and azaserine) for the given enzyme. However, to the best of our knowledge, there is no report so far correlating these differences among various GGTs, to it’s cavity structure and environment, although an excellent review was recently put across in form of a book chapter by the veterans of GGT field: “Gamma-Glutamyl transpeptidase: Structure and function” of “Springer Briefs in Biochemistry and Molecular Biology” (DOI:*10.1007/978-3-0348-0682-4_1*) which is included as reference no. 34 in the manuscript. This chapter enumerates in detail the variety of differences seen in the enzymatic activity of different GGTs and has served as the starting point for most of our work on GGT, where we are attempting to correlate the structural and functional differences seen among various GGTs. Correlating the enormous number of mutations reported earlier with the structure of binding cavity has not been quick and easy, especially because GTT is a bi-substrate enzyme (donor substrate and acceptor substrate) plus the fact that the acceptor site is not well defined till now. We are in the process of comparing the structures of binding cavities of various GGT proteins reported so far and hope to be able to report some correlations soon, but have refrained from reporting any makeshift observations in this manuscript.*

3. On page 13 in Methods the authors list several sequence alignment programs that are tried and also admit to "some manual editing in Bioedit". Since their analysis is restricted to enzymes with solved structures then a sequence alignment based on a structural alignment would really be the gold standard and, indeed, the sequences are quite diverse so it is surprising that they have not tried to do this.

**Author’s response:***Yes, we sure could have tried that. We now tried “TCoffee Expresso” for all forty seven GGT proteins sequences using information of the structures available in PDB (Additional file**5**: Figure S4). The results of this alignment when compared with the sequence alignment used in the manuscript earlier show no major difference, specifically none with respect to the sites discussed in the paper. Probably the sequences are similar enough to generate a respectable alignment even though the proteins exhibit functional variability. This again suggests to us that small differences in these sequences may be responsible for this variability.*

4. In the caption for Table 2 the authors state two levels of significance (*P* < 0.05 and *P* < 0.01). I'm not sure why they don't just state significance at *P* < 0.01.

**Author’s response:***Yes, we totally agree that reporting significance at P < 0.01 should have been enough. It has been an oversight, where the output of the program (which reports at two level of significances P < 0.05 and P < 0.01) has been included ditto into the manuscript. The corrections have been made and incorporated in manuscript.*

5. 22 of the 47 NCBI gi codes given in supp1.doc are obsolete.

**Author’s response:** The *NCBI gi numbers of all 47 GGT sequences have been carefully checked and replaced with the new gi numbers from the NCBI database.*

6. Only four Figures are referred to in the text and there are only captions for four but there are eight Figures in the version of the manuscript that I have and the extra figures look potentially interesting.

**Author’s response:***Actually the last two figures are sets of three figures each, Figs.**3**(a, b and c) and Figs.**4**(a, b and c) but it appears that during uploading of these figures, they were automatically segregated as figure number 3, 4, 5, 6, 7, and 8, even though they were marked as 3a, 3b, 3c, 4a, 4b and 4c. In the revised manuscript the changes have been edited again.*

**Quality of written English:** Acceptable

### Reviewer 2, second report (Christine Orengo, University College London, United Kingdom)

The authors have provided satisfactory responses to address all my concerns and I would be happy for the paper to be published now. However, I do think they should make the rationale for their selection of sequences for the analysis much more explicit in their methods section especially since all the reviewers were confused and concerned by this. There is still not much detail in the methods section. I think they could just add some of the text that they wrote in their response to reviewer 1 ie they should explain why they don’t use all the sequences available via PSIBLAST or Pfam because the set is then too diverse so it doesn’t give a stable enough tree and the alignments are problematic and make it difficult to identify the conserved residues in the different branches of the tree being analyzed. They should also specify the cut-off that they use with BLAST when identifying close homologues. I could not find this.

**Quality of written English:** Acceptable

**Author’s response:***We thank the reviewer for her positive reception. As per the reviewer’s suggestion, more details of sequence selection criteria are now incorporated in text (methodology section, first paragraph).*

### Reviewer 3, first report (Dr. Srikrishna Subramanian, Institute of Microbial Technology, India)

In the paper entitled, “Phylogenetic and evolutionary analysis of functional divergence among Gamma glutamyl transpeptidase (GGT) sub-families”, Verma et al, based on the analysis of 47 GGT sequences argue for the evolutionary divergence of this family into two major branches, with a total of four clades specific to eukaryotes, archaea, pathogenic and non-pathogenic bacteria. They further rationalize clade-specific differences in GGT based on sequence conservation, selection, and functional divergence. Apart from reiterating the importance of the catalytic and binding-site residues for function the study identifies clade-specific changes that may be involved in conferring functional divergence. In addition, they highlight the plausible importance of the large/heavy subunit of GTT in function. The following concerns, if addressed, could likely help improve the manuscript.

**Author’s response:***We thank the reviewer for his valuable suggestions to improve the manuscript.*

**Major concerns:**

1. The GGT family of the Pfam database (PF01019) contains more than 20,000 sequences. The authors however use only 47 GTT sequences for all their analysis. It is not clear from reading the manuscript as to how the authors arrived at this set of sequences and if it is an unbiased dataset that represents the diversity of the GTT family. A larger subset, selected by clustering all sequences of the GTT family (PF01019) could possibly provide more robust results.

**Author’s response:***We think part of this question has been answered before (as part of response to query by reviewer 1). We fully agree that the 47 sequences do not take into account the complete diversity of the GGT sequences found in all organism, but we would only like to reiterate that the objective of the present work is not just to explore complete diversity among GGT sequences but to correlate some of the observed sequence diversity to functional variability. This makes it necessary for us to use sequences which have been biochemically characterized earlier as seed sequences. We then find homologs that are close to these seed sequences for amplifying the signal of similarity. The homologs that are used to make this alignment have to be pretty close to the seed sequence because we have to be confident of them having similar biochemical characteristics as the seed sequence. Only then can we possibly correlate the similarities and differences among two clades to be responsible for functional differences. Adding sequences that are too different does increase diversity but dilutes the signal of amino acid conservation and since no structural or biochemical information is available for most of these GGT protein sequences, the correlation cannot be established.*

2. It is not clear after reading the introduction as to which residues are involved in the enzymatic reaction and what the mechanism is? A schematic diagram of the enzymatic reaction together with a short description of the structure, catalytic mechanism and functionally-important residues from each subunit should be provided.

**Author’s response:***Following reviewer’s advice,* a *short paragraph describing the enzymatic mechanism of GGT has now been included in the “Background” as 3*^*rd*^*para. In GGT enzymes, three conserved residues are directly involved in the reaction mechanism. The conserved N-terminal threonine (Thr) residue of the small subunit acts as nucleophile and it is involved in both the autoprocessing and the glutathione catalysis. The other two conserved glycine residues (Gly-Gly) are known as oxyanion hole residues, play a key role in stabilization of gamma-glutamyl-GGT enzyme intermediate complex. A schematic diagram (Additional file**1**) of GGT enzyme mechanism has been incorporated in the background section of the manuscript. Also Table*1*in the manuscript enumerates and compares residues that have been shown to be part of the binding cavity and are known to interact with the substrate and influence the enzyme activity in GGTs from various organisms.*

3. The sequence motif-conservation logos shown in the manuscript are misleading as they are based on the conservation-patterns of only 47 sequences and do not take into account the diversity of the GTT family. For example, in Motif1, E. coli Asp75 is shown to be absolutely conserved, but if we look at the HMM profile of GTT family in Pfam, this Asp is highly but not absolutely conserved.

***Author’s response:****In continuation of our response to Major Concern 1 by the same reviewer, we believe that small differences in the conservation of individual amino acids in 47 sequences versus 20000 sequences of Pfam should not affect the analysis in any significant way since the objective here is to compare two well separated clades which are known to exhibit functional variability.*

4. The numbers reported in the Tables and Additional files need to be rounded off in a consistent manner. Currently the statistical-values for attributes in the results for selection and divergence analysis are shown from one to six decimal places? How is this meaningful?

**Author’s response:***In Tables* (2, 3) *and Additional file**3**, all values are original, which were achieved in statistical calculations of positive selection and functional divergence analysis using various softwares and were used as such. At the suggestion of the reviewer, these values in tables and additional files have now been rounded off to the second place of decimal to maintain consistency.*

**Minor concerns:**

1. The manuscript is difficult to read and needs to be edited carefully. Many places have inappropriate use of CAPS in words. For e.g. ‘Codeml’ and ‘Codeml’ seem to be used interchangeably. Please look for similar errors.

**Author’s response:***In light of the reviewer’s comment, the manuscript has been carefully revised and all similar errors are now fixed.*

2. Scientific names should be written as per standard convention. for e.g. please correct has E. Coli, H. Pylori, etc. in the 3rd paragraph of the Background section. There are several such errors that need to be fixed.

**Author’s response:***All similar errors have been corrected and incorporated in the manuscript.*

3. The references for the programs used for data analysis are provided only in the Methods section. They need to be provided at the first instance they are mentioned. For example in the first line of the Functional divergence in GGT-family section, Codeml is mentioned without a citation and is cited only later in the manuscript.

**Author’s response:***This has been corrected and all programs are now cited with suitable references at the first instance.*

4. Consistent use of either one letter or three letter codes of the amino acids could improve readability.

**Author’s response:***All changes have been incorporated and amino acids are now denoted by single letter code.*

5. The use of the terms larger and smaller subunits is awkward at times. For example in the Results section "The larger subunit has largely been neglected…". Why not call this the large and small subunit or better still the heavy and light subunits as mentioned previously in literature.

**Author’s response:***All changes have been made and the terms “small” and “large” subunit are now used consistently throughout the manuscript.*

6. Please provide PDB identifiers for the structurally characterized GGTs when those are referred. For example, in the background section when the authors refer to “the three dimensional structures of GGTs from varied organisms, including human GGT1, E. coli, H. pylori, B. subtilis, B. halodurans and T. acidophilum, with and without substrate analogues”.

**Author’s response:***All information regarding the structures of GGT deposited in PDB databases is now summarized in tabular form and incorporated as Additional file**2**of the supplementary material.*

7. The legend for figures 3 and 4 corresponds to figures 3/4/5 and figures 6/7/8, respectively. These figures need to be compiled into a single figure 3 and figure.

**Author’s response:***Actually the last two figures are sets of three figures each, Fig.**3**(**a*, *b** and **c**) and Fig.**4**(**a*, *b** and **c**) but it appears that during uploading of these figures, they were automatically segregated as figure number 3, 4, 5, 6, 7, and 8, even though they were marked as 3a, 3b, 3c, 4a, 4b and 4c. In the revised manuscript the changes have been edited again.*

8. Further the structural figures are too cluttered to follow. I would suggest using a program like Ligplot to show these residues.

**Author’s response:***Following reviewer suggestion, we tried LIGPLOT but it seems to be unavailable at the moment. In our experience, LIGPLOT is especially helpful in highlighting residues interacting with the substrate, but in the current setting, we needed to represent the overall spread of the residues that have been predicted through DIVERGE program to be involved in functional divergence (ex Figs.**3b**and**4b**). This figure is meant to convey the higher concentration of such putative sites near the binding cavity of GGT. This is again emphasized through Figs.**3c** and**4c** which marks only residues that are part of the binding site, and among these, the ones that are predicted to be involved in functional divergence are marked in red, suggesting that quite a few residues that are forming the cavity can be statistically predicted to be contributing to functional divergence.*

**Quality of written English:** Needs some language corrections before being published.

**Author’s response:***The manuscript has been revised and corrections have been made.*

### Reviewer 3, second report (Dr. Srikrishna Subramanian, Institute of Microbial Technology, India)

I don't have any major comments. There is a typo in the spelling of transpeptidation in the last line of the Additional file 1.

**Quality of written English:** Acceptable

**Author’s response:***We thank the reviewer for his positive response. Suggested changes have been incorporated in Additional file**1**.*

## Additional files

Additional file 1: Figure S1. Proposed GGT mechanism: Figure represents the schematic flow diagram for catalysis of γ-glutamyl tripeptide (glutathione) cleavage by GGT. In first step, N-terminal Thr residue of small subunit attacks on γ-glutamyl peptide bond of glutathione (GSH). Second step leads to formation of transition state. In third step, ‘Cys-Gly’ is released from the glutathione and forms a γ-glutamyl-GGT complex. Forth step involves the transfer of its γ-glutamyl moiety to water molecule or short peptide or amino acids and gives either hydrolysis or transpeptidation reaction. (DOC 214 kb) Additional file 2: Table S1. Detailed list of PDB identifiers for structurally characterized GGT proteins deposited in Protein Databank. (DOC 65 kb) Additional file 3: Figure S2. Detailed multiple sequence alignment GGT family (47 GGT sequences). Detailed list of GGT gene ids (shown in closed brackets) along with organism names used in comparative analysis. (DOC 650 kb) Additional file 4: Table S2. Comparative divergence analysis type I and type II in distinct GGT clades (DOC 73 kb) Additional file 5: Figure S3. Phylogenetic tree of GGT proteins including non pathogenic proteobacteria. **Figure S4.** Structure based sequence alignment of GGT proteins. In the given figure, on the top of the row secondary structure elements of the GGT proteins have been shown with their respective numbering. GGT proteins are highly conserved in secondary structure pattern and shared αβ_1_β_2_α sandwich like protein folds. The alpha helical and β-strands regions of GGT proteins are represented by α and β notations respectively whereas remaing part of the aligned proteins might contain loop and coil regions. All secondary structure fragments are generated by using 3D structure information of *E. coli* GGT (2E0W). The 3D structure based sequence alignment is performed by using “TCoffee Expresso” server (http://tcoffee.crg.cat/apps/tcoffee/do:expresso) and final alignment figure is generated by using ESPript3.0 (http://espript.ibcp.fr/ESPript/ESPript/) online available tools. Highly conserved residues are hilighted in red color shadow. (DOC 7750 kb)

## References

[1] Tate SS, Meister A (1981). γ-Glutamyl transpeptidase: catalytic, structural and functional aspects. Mol Cell Biochem.

[2] Allison RD (1985). Gamma-Glutamyl transpeptidase: kinetics and mechanism. Methods Enzymol.

[3] Keillor JW, Castonguay R, Lherbet C (2005). Gamma-glutamyl transpeptidase substrate specificity and catalytic mechanism. Methods Enzymol.

[4] Heisterkamp N, Ven JG, Warburton D, Sneddon TP (2008). The human gamma-glutamyltransferase gene family. Human Genetics.

[5] Hanigan MH, Ricketts WA (1993). Extracellular glutathione is a source of cysteine for cells that express gamma-glutamyl transpeptidase. Biochemistry.

[6] Mehdi K, Penninckx MJ (1997). An important role for glutathione and gamma-glutamyltranspeptidase in the supply of growth requirements during nitrogen starvation of the yeast Saccharomyces cerevisiae. Microbiology.

[7] Hinchman CA, Ballatori N (1994). Glutathione conjugation and conversion to mercapturic acids can occur as an intrahepatic process. J Toxicol Environ Health.

[8] Meister A, Tate SS (1976). Glutathione and related gamma-glutamyl compounds: biosynthesis and utilization. Annu Rev Biochem.

[9] Ritz D, Beckwith J (2001). Roles of thiol-redox pathways in bacteria. Annu Rev Microbiol.

[10] Suthanthiran M, Anderson ME, Sharma VK, Meister A (1990). Glutathione regulates activation-dependent DNA synthesis in highly purified normal human T lymphocytes stimulated via the CD2 and CD3 antigens. Proc Natl Acad Sci U S A.

[11] Vergauwen (2001). Characterization of glutathione amide reductase from Chromatium gracile. Identification of a novel thiol peroxidase (Prx/Grx) fueled by glutathione amide redox cycling. J Biol Chem.

[12] Wang W, Ballatori N (1998). Endogenous glutathione conjugates: occurrence and biological functions. Pharmacol Rev.

[13] Inoue M, Hiratake J, Suzuki H, Kumagai H, Sakata K (2000). Identification of catalytic nucleophile of Escherichia coli gamma-glutamyltranspeptidase by gamma-monofluorophosphono derivative of glutamic acid: N-terminal thr-391 in small subunit is the nucleophile. Biochemistry.

[14] Minami H, Suzuki H, Kumagai H (2004). γ-Glutamyltranspeptidase, but not YwrD, is important in utilization of extracellular glutathione as a sulfur source in *Bacillus subtilis*. J Bacteriol.

[15] Okada T, Suzuki H, Wada K, Kumagai H, Fukuyama K (2007). Crystal structure of the gamma-glutamyltranspeptidase precursor protein from Escherichia coli. Structural changes upon autocatalytic processing and implications for the maturation mechanism. J Biol Chem.

[16] Hu X (2012). Probing the donor and acceptor substrate specificity of the γ-Glutamyl transpeptidase. Biochemistry.

[17] Okada T, Suzuki H, Wada K, Kumagai H, Fukuyama K (2006). Crystal structures of γ-glutamyltranspeptidase from Escherichia coli, a key enzyme in glutathione metabolism, and its reaction intermediate. Proc Natl Acad Sci USA.

[18] Schulman JD, Goodman SI, Mace JW, Patrick AD, Tietze F, Butler EJ (1975). Glutathionuria: inborn error of metabolism due to tissue deficiency of gamma-glutamyl transpeptidase. Biochem Biophys Res Commun.

[19] Wright EC, Stern J, Ersser R, Patrick AD (1979). Glutathionuria: gamma-glutamyl transpeptidase deficiency. J Inherit Metab Dis.

[20] Castellano I, Merlino A (2012). γ-Glutamyltranspeptidases. Sequence, structure, biochemical properties, and biotechnological applications. Cell Mol Life Sci.

[21] Betro MG, Oon RC, Edwards JB (1973). Gamma-glutamyl transpeptidase in diseases of the liver and bone. Am J Clin Pathol.

[22] Owen AD, Schapira AH, Jenner P, Marsden CD (1996). Oxidative stress and Parkinson’s disease. Proc Natl Acad Sci USA.

[23] Mena S (2007). Bcl-2 and glutathione depletion sensitizes B16 melanoma to combination therapy and eliminates metastatic disease. Clin Cancer Res.

[24] Richter S (2009). Capsule anchoring in Bacillus anthracis occurs by a transpeptidation reaction that is inhibited by capsidin. Mol Microbiol.

[25] Marshall BJ, Warren JR (1984). Unidentified curved bacilli in the stomach of patients with gastritis and peptic ulceration. Lancet.

[26] Chevalier C, Thiberge JM, Ferrero RL, Labigne A (1999). Essential role of Helicobacter pylori gamma glutamyltranspeptidase for the colonization of the gastric mucosa of mice. Mol Microbiol.

[27] Schmees C (2007). Inhibition of T-cell proliferation by Helicobacter pylori gamma-glutamyl transpeptidase. Gastroenterology.

[28] King JB, West MB, Cook PF, Hanigan MH (2008). A novel, species-specific class of uncompetitive inhibitors of γ-Glutamyl transpeptidase. J Biol Chem.

[29] Boanca G, Sand A, Okada T, Suzuki H, Kumagai H, Fukuyama K, Barycki JJ (2007). Autoprocessing of *Helicobacter pylori* γ-Glutamyltranspeptidase leads to the formation of a threonine-threonine catalytic dyad. J Biol Chem.

[30] Wada K (2008). Crystal structures of Escherichia coli γ-glutamyltranspeptidase in complex with azaserine and acivicin: Novel mechanistic implication for inhibition by glutamine antagonists. J Mol Biol.

[31] Wada K, Irie M, Suzuki H, Fukuyama K (2010). Crystal structure of the halotolerant gamma glutamyltranspeptidase from *Bacillus subtilis* in complex with glutamate reveals a unique architecture of the solvent-exposed catalytic pocket. FEBS J.

[32] Castellano I, Merlino A, Rossi M, La Cara F (2010). Biochemical and structural properties of gamma-glutamyl transpeptidase from Geobacillus thermodenitrificans: an enzyme specialized in hydrolase activity. Biochimie.

[33] Castellano I, Di Salle A, Merlino A, Rossi M, La Cara F (2011). Gene cloning and protein expression of γ-glutamyltranspeptidases from Thermus thermophilus and Deinococcus radiodurans: comparison of molecular and structural properties with mesophilic counterparts. Extremophiles.

[34] Rajput R, Verma VV, Chaudhary V, Gupta R (2013). A hydrolytic γ-glutamyl transpeptidase from thermo-acidophilic archaeon Picrophilus torridus: binding pocket mutagenesis and transpeptidation. Extremophiles.

[35] West MB, Chen Y, Wickham S, Herous A, Cahill K, Hanigan MH, Mooers BHM (2013). Novel Insights into Eukaryotic γ-Glutamyltranspeptidase 1 from the Crystal Structure of the Glutamate-bound Human enzyme. J Biol Chem.

[36] Terzyan SS, Burgett AWG, Heroux A, Smith CA, Mooers BHM, Hanigan MH (2015). Human -Glutamyl Transpeptidase 1: Structures of the free enzyme, inhibitor-bound tetrahedral transition states, and glutamate-bound enzyme reveal novel movement within the active site during catalysis. J Biol Chem.

[37] Lin LL, Chen YY, Chi MC, Merlino A (2014). Low resolution X-ray structure of γ-glutamyltranspeptidase from Bacillus licheniformis: opened active site cleft and a cluster of acid residues potentially involved in the recognition of a metal ion. Biochim Biophys Acta.

[38] Wu R (2009). Crystal Structure of Bacillus anthracis transpeptidase enzyme CapD. J Biol Chem.

[39] Bailey TL (2009). MEME SUITE: tools for motif discovery and searching. Nucleic Acids Res.

[40] Warshel A (1978). Energetics of enzyme catalysis. Proc Natl Acad Sci USA.

[41] Warshel A, Sussman F, Hwang JK (1988). Evaluation of catalytic free energies in genetically modified proteins. J Mol Biol.

[42] Yang Z (1998). Likelihood ratio tests for detecting positive selection and application to primate lysozyme evolution. Mol Biol Evol.

[43] Li W, Jaroszewski L, Godzik A (2001). Clustering of highly homologous sequences to reduce the size of large protein database. Bioinformatics.

[44] Notredame C, Higgins DG, Heringa J (2000). T-Coffee: A novel method for fast and accurate multiple sequence alignment. J Mol Biol.

[45] Larkin MA (2007). Clustal W and Clustal X version 2.0. Bioinformatics.

[46] Edgar RC (2004). MUSCLE: multiple sequence alignment with high accuracy and high throughput. Nucleic Acids Res.

[47] Hall TA (1999). Bioedit: a user friendly biological sequence alignment editor and analysis program for Windows 95/98/NT. Nucleic Acids Symp Ser.

[48] Tamura K, Peterson D, Peterson N, Stecher G, Nei M, Kumar S (2011). MEGA5: Molecular Evolutionary Genetics Analysis Using Maximum Likelihood, Evolutionary Distance, and Maximum Parsimony Methods. Mol Biol Evol.

[49] Darriba D, Taboada GL, Doallo R, Posada D (2011). ProtTest 3: fast selection of best-fit models of protein evolution. Bioinformatics.

[50] Liang GM, Jiang XP (2010). Positive selection drives lactoferrin evolution in mammals. Genetica.

[51] Guindon S, Gascuel O (2003). A simple, fast, and accurate algorithm to estimate large phylogenies by maximum likelihood. Syst Biol.

[52] Yang Z (2007). PAML 4: a program package for phylogenetic analysis by maximum likelihood. Mol Biol Evol.

[53] Nei M, Gojobori T (1986). Simple methods for estimating the numbers of synonymous and nonsynonymous nucleotide substitutions. Mol Biol Evol.

[54] Goldman N, Yang Z (1994). A codon-based model of nucleotide substitution for protein-coding DNA sequences. Mol Biol Evol.

[55] Yang Z, Nielsen R (1998). Synonymous and nonsynonymous rate variation in nuclear genes of mammals. J Mol Evol.

[56] Yang Z, Nielsen R, Goldman N, Pedersen AMK (2000). Codon-substitution models for heterogeneous selection pressure at amino acid sites. Genetics.

[57] Gu X, Zou Y, Su Z, Huang W, Zhou Z, Arendsee A, Zeng Y (2013). An update of DIVERGE software for functional divergence analysis of protein family. Mol Biol Evol.

[58] Gu X (1999). Statistical methods for testing functional divergence after gene duplication. Mol Biol Evol.

[59] Gu X (2001). Maximum-likelihood approach for gene family evolution under functional divergence. Mol Biol Evol.

[60] Gu X (2001). A site-specific measure for rate difference after gene duplication or speciation. Mol Biol Evol.

[61] Gu X (2003). Functional divergence in protein (family) sequence evolution. Genetica.

[62] Gu X (2006). A simple statistical method for estimating type-II (cluster specific) functional divergence of protein sequences. Mol Biol Evol.

[63] Seeliger D, De Groot BL (2010). Ligand docking and binding site with PyMOL and Autodock/Vina. J Comput Aided Mol Des.

