# Supplementary material for: “Phylogenetic and evolutionary analysis of functional divergence among Gamma glutamyl transpeptidase (GGT) subfamilies”
Source: Biol Direct. 2015 Sep 14;10:49. doi: 10.1186/s13062-015-0080-7 (PMC4568574; doi:10.1186/s13062-015-0080-7)
Supplement: Additional file 1: Figure S1. — Proposed GGT mechanism: Figure represents the schematic flow diagram for catalysis of γ-glutamyl tripeptide (glutathione) cleavage by GGT. In first step, N-terminal Thr residue of small subunit attacks on γ-glutamyl peptide bond of glutathione (GSH). Second step leads to formation of transition state. In third step, ‘Cys-Gly’ is released from the glutathione and forms a γ-glutamyl-GGT complex. Forth step involves the transfer of its γ-glutamyl moiety to water molecule or short peptide or amino acids and gives either hydrolysis or transpeptidation reaction. (DOC 214 kb) [file 13062_2015_80_MOESM1_ESM.doc]

**Additional file 1**

**Figure S1: Proposed GGT mechanism**


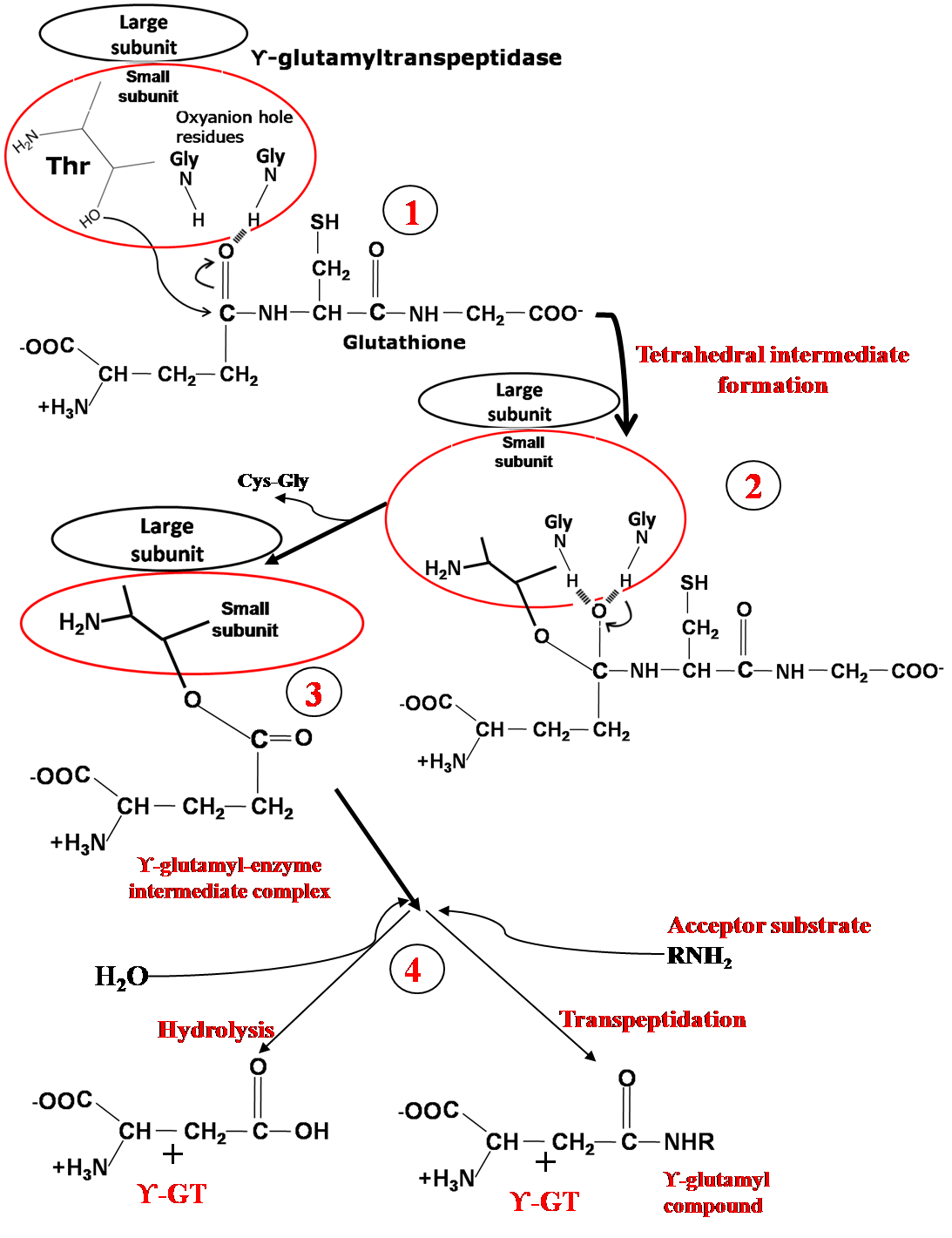


Figure represents the schematic flow diagram for catalysis of γ-glutamyl tripeptide (glutathione) cleavage by GGT. In first step, N-terminal Thr residue of small subunit attacks on γ-glutamyl peptide bond of glutathione (GSH). Second step leads to formation of transition state. In third step, ‘Cys-Gly’ is released from the glutathione and forms a γ-glutamyl-GGT complex. Forth step involves the transfer of its γ-glutamyl moiety to water molecule or short peptide or amino acids and gives either hydrolysis or transpeptidation reaction.
