# Supplementary material for: “Phylogenetic and evolutionary analysis of functional divergence among Gamma glutamyl transpeptidase (GGT) subfamilies”
Source: Biol Direct. 2015 Sep 14;10:49. doi: 10.1186/s13062-015-0080-7 (PMC4568574; doi:10.1186/s13062-015-0080-7)
Supplement: Additional file 2: Table S1. — Detailed list of PDB identifiers for structurally characterized GGT proteins deposited in Protein Databank. (DOC 65 kb) [file 13062_2015_80_MOESM2_ESM.doc]

**Additional file 2**

**Table S1:**

**Detailed list of PDB identifiers for structurally characterized GGT proteins deposited in Protein Databank**

| **Organism** | **PDB code** | **Resolution** | **Structure description** | **Metal ion** | **PubMed id** |
| --- | --- | --- | --- | --- | --- |
| *Escherichia*  *coli* | 2DBU | 1.95 Å | Ligand free form | Not bound | 16618936 |
|  | 2DG5 | 1.60 Å | Complex with hydrolyzed glutathione | Ca^2+^ | 16618936 |
|  | 2Z8K | 1.65 Å | Complex with acivicin | Not bound | 18555071 |
|  | 2DBX | 1.70 Å | Complex with L-glutamate | Ca^2+^ | 16618936 |
|  | 2Z8I | 1.65 Å | Complex with azaserine | Not bound | 18555071 |
|  | 2DBW | 1.80 Å | Acyl-enzyme intermediate | Not bound | 16618936 |
|  | 2E0X | 1.95 Å | Monoclinic form | Ca^2+^ | 17135273 |
|  | 2E0Y | 2.02 Å | Samarium derivative | Sm^3+^ | 17135273 |
|  | 2Z8J | 2.05 Å | Complex with azaserine prepared in the dark | Not bound | 18555071 |
|  | 2E0W | 2.55 Å | T391A mutant | Not bound | 17135273 |
| *Helicobacter*  *pylori* | 2NQO | 1.90 Å | Ligand free form | Not bound | 17107958 |
|  | 2QM6 | 1.60 Å | Complex with glutamate | Not bound | 17960917 |
|  | 3FNM | 1.70 Å | Complex with acivicin | Not bound | 19256527 |
|  | 2QMC | 1.55 Å | Mature T380A mutant in complex with S(nitrobenzyl)glutathione | Not bound | 17107958 |
| *Bacillus*  *subtilis* | 2V36 | 1.85 Å | Ligand free form | Not bound | To be published |
|  | 3A75 | 1.95 Å | Complex with glutamate | Not bound | 20088880 |
| *Bacillus licheniformis* | 4OTT | 2.98 Å | Ligand free form | Not bound | To be published |
|  | 4OTU | 3.02 Å | Complex with L-glutamate | Mg^2+^ | 24780583 |
| *Thermoplasma*  *acidophilum* | 2I3O | 2.03 Å | Ligand free form | Not bound | To be published |
| *Bacillus*  *haloduran* | 2NLZ | 2.70 Å | Ligand free form | Not bound | To be published |
| Human  (GGT1) | 4GDX | 1.67 Å | Complex with glutamic acid | Cl^-^, Na^+^ | 24047895 |
|  | 4GG2 | 2.21 Å | Complex with glutamic acid | Cl^-^, I^-^ | 24047895 |
|  | 4Z9O | 2.30 Å | Ligand free form | Cl^-^, Na^+^ | 26013825 |
|  | 4ZC6 | 2.10 Å | Complex with Serine Borate | Cl^-^, Na^+^ | 26013825 |
|  | 4ZBK | 2.18 Å | Complex with GGs Top inhibitor | Cl^-^, Na^+^ | 26013825 |
|  | 4ZCG | 2.22 Å | Complex with glutamate | Cl^-^, Na^+^ | 26013825 |
| *Bacillus anthracis* | 3G9K | 1.79 Å | Complex with glutamic acid | Not bound | 19535342 |
|  | 3GA9 | 2.30 Å | Complex with glutamic acid | Not bound | 19535342 |

Sm^3+^ = Samarium (III) ion

Ca^2+^  = Calcium (II) ion

Cl^-^ = Chlorine ion

I^-^ = Iodine ion

Na^+^ = Sodium ion

Mg^2+^  = Magnesium ion
