# Supplementary material for: “Phylogenetic and evolutionary analysis of functional divergence among Gamma glutamyl transpeptidase (GGT) subfamilies”
Source: Biol Direct. 2015 Sep 14;10:49. doi: 10.1186/s13062-015-0080-7 (PMC4568574; doi:10.1186/s13062-015-0080-7)
Supplement: Additional file 3: Figure S2. — Detailed multiple sequence alignment GGT family (47 GGT sequences). Detailed list of GGT gene ids (shown in closed brackets) along with organism names used in comparative analysis. (DOC 650 kb) [file 13062_2015_80_MOESM3_ESM.doc]

**Additional file 3**

**Figure S2:**

**Detailed multiple sequence alignment GGT family (47 GGT sequences)**


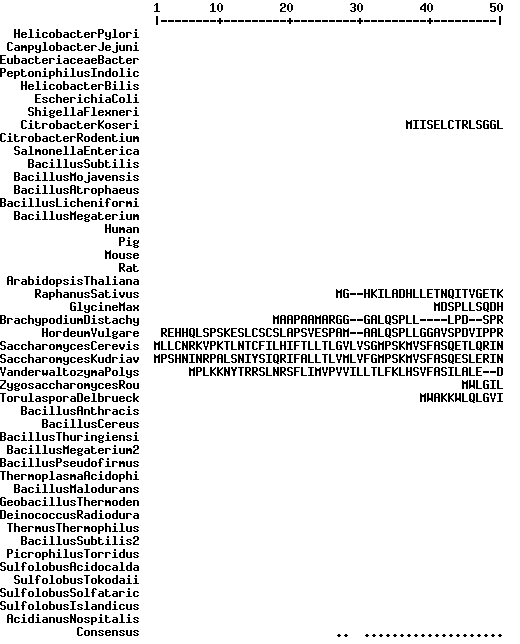


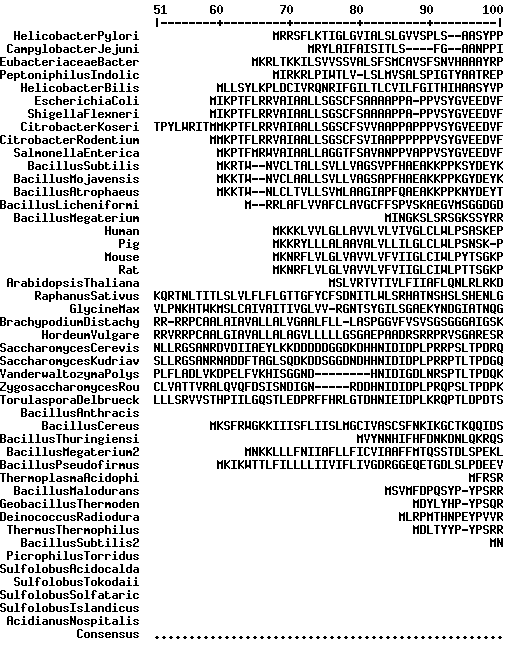


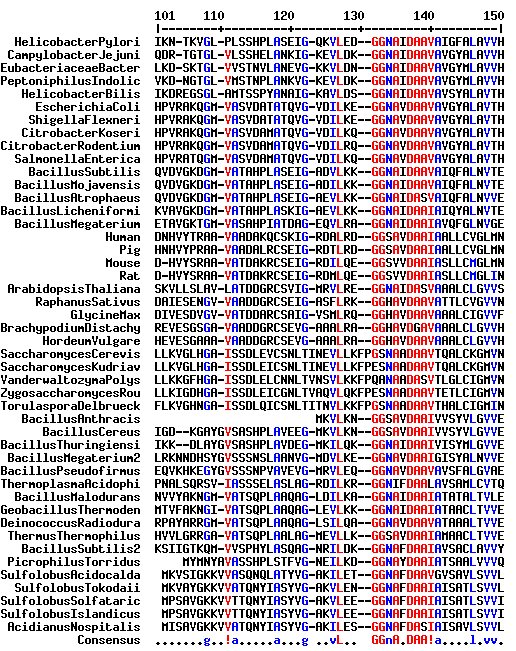


**MOTIF M1**

**GGXXXXDAAI/V**


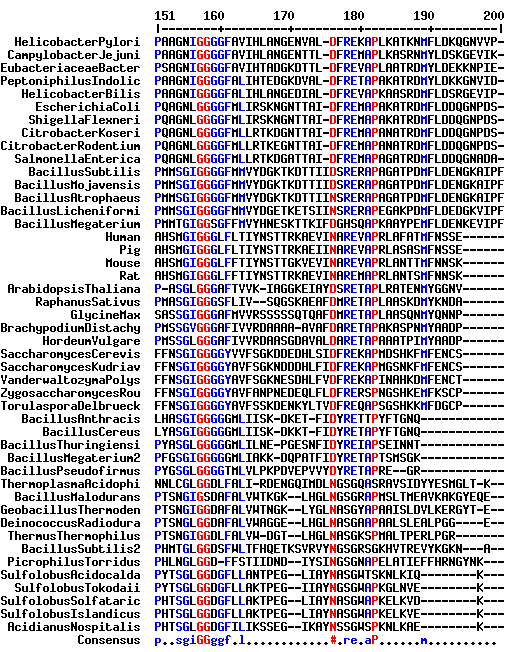


**MOTIF M1**


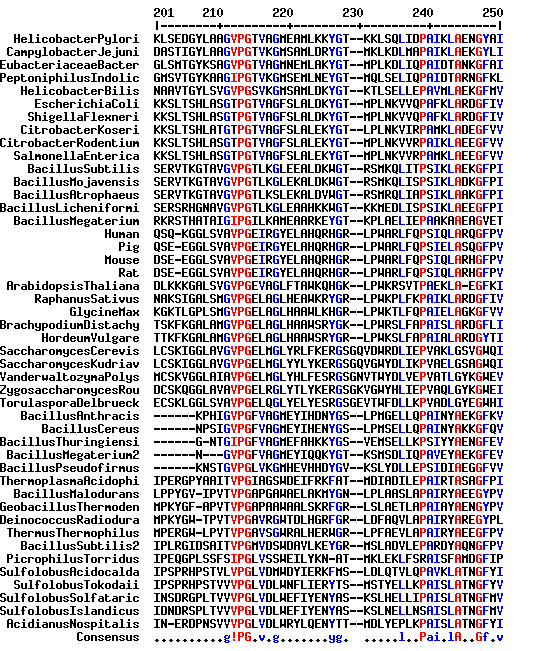


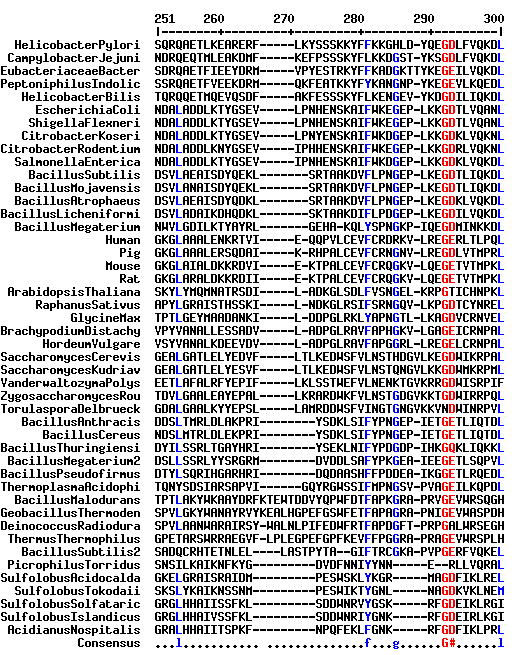
\


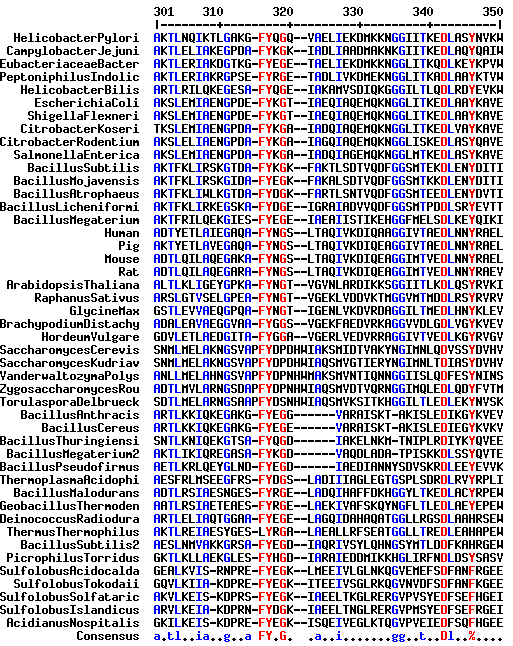


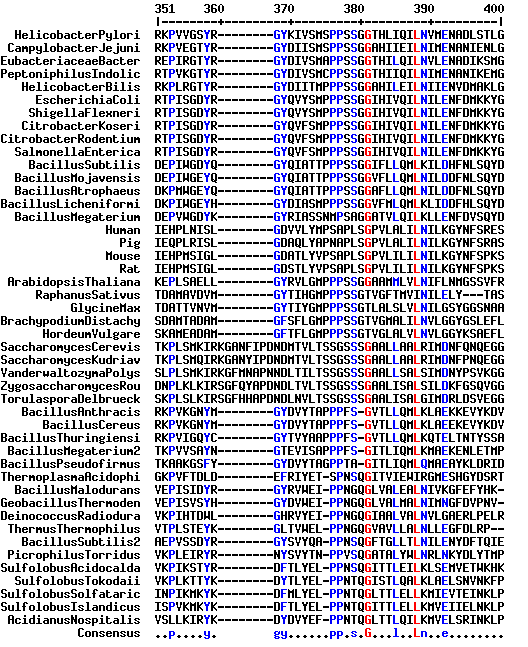


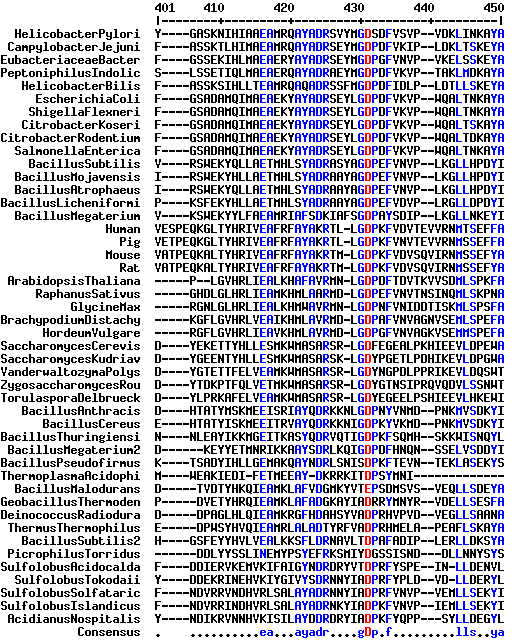


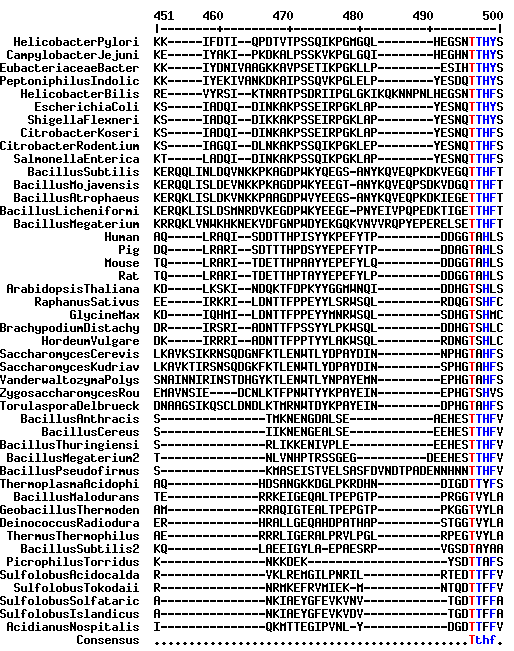


**N-terminal nucleophile, red T**

**MOTIF M2**

**TXX**

**MOTIF M2**


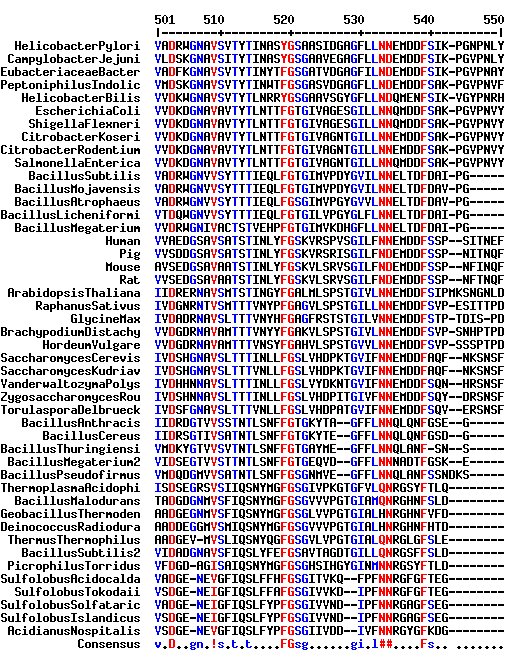


**TXN/TXN/SXY/SXF**

**Conserved GXXGG motif**

**MOTIF M3**


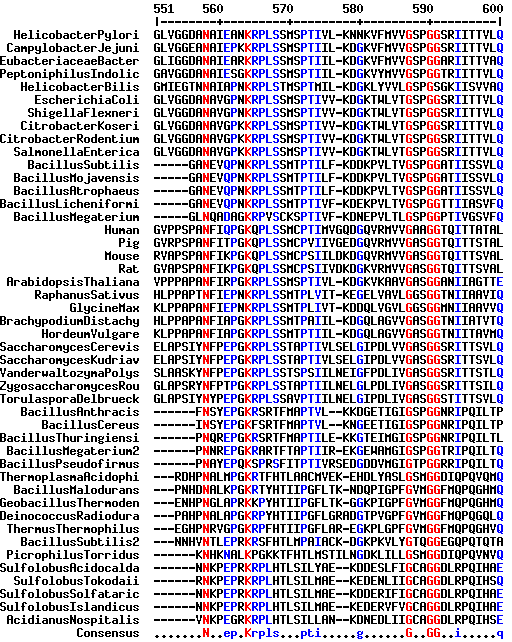


**LSSMSP/LSSXXX**


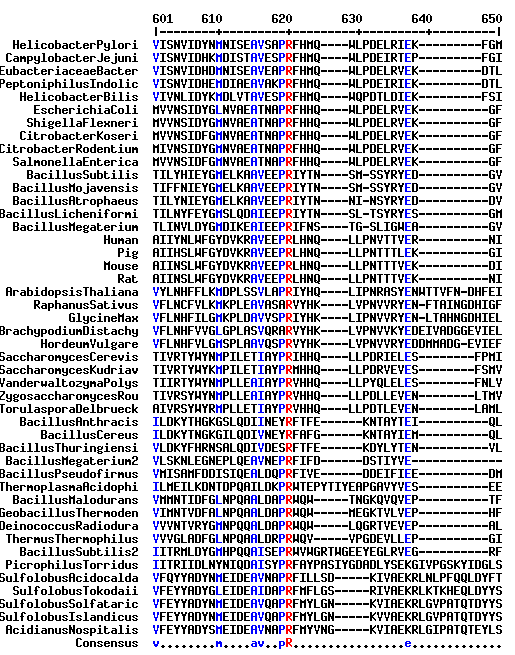


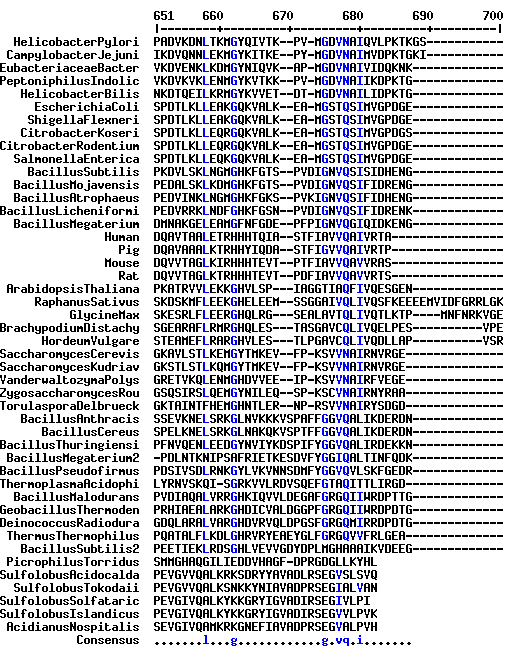


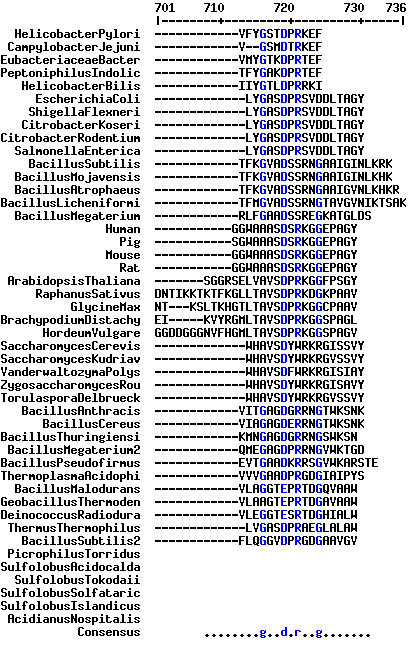


**Detailed list of GGT gene ids (shown in closed brackets) along with organism names used in comparative analysis:**

1. Helicobacter pylori (11321265)

2. Campylobacter jejuni (498489045)

3. Eubacteriaceae bacterium (497216078, Peptostreptococcaceae bacterium)

4. Peptoniphilus indolicus (490957348)

5. Helicobacter bilis (229373893)

6. Escherichia coli (146133)

7. Shigella flexneri (446517734)

8. Citrobacter koseri (157086234)

9. Citrobacter rodentium (502672509)

10. Salmonella enterica (446727777)

11. Bacillus anthracis (436034)

12. Bacillus pseudofirmus (502725675)

13. Bacillus megaterium (502820735)

14. Bacillus thuringiensis (489384092)

15. Bacillus cereus (487922716)

16. Bacillus subtilis (407959255)

17. Bacillus licheniformis (499511173)

18. Bacillus mojavensis (498020308)

19. Bacillus atrophaeus (489420259)

20. Bacillus megaterium (504273729)

21. Thermoplasma acidophilum (10640309)

22. Picrophilus torridus (48430905)

23. Bacillus licheniformis (489278186)

24. Saccharomyces cerevisiae (596034)

25. Saccharomyces kudriavzevii (401840184)

26. Zygosaccharomyces rouxii (254582188)

27. Vanderwaltozyma polyspora (156840692)

28. Torulaspora delbrueckii (367016909)

29. Human (73915092)

30. Mouse (2494732)

31. Arabidopsis thaliana (928934)

32. Raphanus sativus (47971189)

33. Brachypodium distachyon (357127344)

34. Glycine max (356520836)

35. Hordeum vulgare (326489959)

36. Sulfolobus acidocaldarius (499596606)

37. Sulfolobus tokodaii (15922686)

38. Sulfolobus solfataricus (497678555)

39. Sulfolobus islandicus (504327036)

40. Acidianus hospitalis (503541804)

41. Bacillus halodurans (10173482)

42. Geobacillus thermodenitrificans (500218239)

43. Deinococcus radiodurans (81551202)

44. Thermus thermophilus (499486211)

45. Rat (145280505)

46. Pig (47522880)

47. Bacillus subtilis (503881428)
