# Supplementary material for: “Phylogenetic and evolutionary analysis of functional divergence among Gamma glutamyl transpeptidase (GGT) subfamilies”
Source: Biol Direct. 2015 Sep 14;10:49. doi: 10.1186/s13062-015-0080-7 (PMC4568574; doi:10.1186/s13062-015-0080-7)
Supplement: Additional file 4: Table S2. — Comparative divergence analysis type I and type II in distinct GGT clades (DOC 73 kb) [file 13062_2015_80_MOESM4_ESM.doc]

**Additional file 4**

**Table S2:**

**Comparative divergence analysis type I and type II in distinct GGT clades**

| **Bacteria 1 / Bacteria 3** | | | | | | **Bacteria 3/ Archaea** | | | | | |
| --- | --- | --- | --- | --- | --- | --- | --- | --- | --- | --- | --- |
| **FD I**  **θI = 0.46±0.06**  **p = 0.67** | | | **FD II**  **θII = 0.02±0.11**  **R = 1.5** | | | **FD I**  **θI = 0.23±0.09**  **p = 0.5** | | | **FD II**  **θII = 0.11±0.10**  **R = 4.0** | | |
| **Site**  302  304  332  351  386  398  433  440  450  486  560  585  602  649  650  675  762 | ***Ec***  E197  L199  T221  D240  A272  R283  N309  G317  A322  N356  T412  K437  A453  I499  D500  D521  A567 | ***Ba***  R179  Y181  T200  K219  K245  M257  K282  V289  Y295  S329  N373  E396  S400  T445  H446  T466  A515 | **Site**  184  187  190  261  295  389  423  543  553  556  610  612  638  640  674 | ***Ec***  A84  H87  A90  L158  D190  R275  H299  S395  A405  Y408  L461  S463  I488  T490  P520 | ***Ba***  G84  E87  A90  H140  R172  R249  T272  V356  S366  N369  R408  F410  P434  I436  K464 | **Site**  362  368  390  547  566  585  602  649  681 | ***Ba***  A227  K232  K250  R360  K379  E396  S400  T445  I471 | ***Sa***  M168  G193  K215  G325  I343  G360  K363  A408  K434 | **Site**  196  213  215  216  295  335  386  408  424  450  541  555  559  610  612  632  636  637  641  656  769 | ***Ba***  G96  D111  R113  E114  R172  Q203  V246  G258  L273  Y295  H357  T368  S372  R408  F410  P428  R432  I433  L437  Q452  G522 | ***Sa***  D55  N70  S72  G73  A138  L164  G211  D223  T238  R260  F319  I332  F336  L371  T374  A392  L395  R396  H400  D415  E472 |

**FDI:** Functional divergence type 1

**P:** Posterior probability

**θI:** Coefficient of divergence type 1

**FDII:** Functional divergence type 2

**R:** Posterior ratio

**θII:** Coefficient of divergence type 2

**Ec:** *Escherichia coli*

**Sa:** *Sulfolobus acidocaldarius*

**Ba:** *Bacillus anthracis*

| **Bacteria 4 extremophile / Archaea** | | | | | | **Bacteria 3 / Eukaryote** | | | | | |
| --- | --- | --- | --- | --- | --- | --- | --- | --- | --- | --- | --- |
| **FD I**  **θI =0.14±0.09**  **p= 0.36** | | | **FD II**  **θII = 0.15±0.09**  **R = 2.0** | | | **FD I**  **θI = 0.53±0.12**  **p = 0.80** | | | **FD II**  **θII = 0.31±0.07**  **R = 4.0** | | |
| **Site**  172  183  199  211  299  333  335  342  376  413  415  546  553  569  577  618  632  640  681  719 | ***Bh***  A47  L58  A74  G84  A166  W199  S201  L208  L239  E266  P268  D365  S372  S383  N396  L426  M439  G447  P483  G510 | ***Sa***  A31  L42  L58  A68  A142  I162  L164  R176  M201  E228  P230  D324  G330  K346  N352  L377  A391  I399  K434  G451 | **Site**  180  211  335  408  429  538  540  551  558  614  625  631  635  637  640  654  655  656  681 | ***Bh***  A55  G85  S201  G261  N281  G357  V359  M370  N377  I422  P432  V438  F442  Q444  G448  N461  P462  Q463  P483 | ***Sa***  S39  A68  L164  D223  K243  D316  T318  E328  L334  S375  E384  C390  D394  R396  I399  E413  I414  D415  K434 | **Site**  163  172  183  199  247  262  302  332  333  335  342  409  489  491  492  546  553  577  585  604  632  640  641  647  686  719  725  756 | ***Ba***  V65  A72  L83  M99  H126  D141  R179  T200  L201  Q203  L210  Y259  Y332  S334  T335  D359  S366  N388  E396  E402  P428  I436  L437  K443  S475  G498  L503  D509 | ***Hm***  A57  A64  V75  L91  S131  Q146  V184  R207  L208  L210  Y217  V272  F350  A352  Q353  A388  S395  N419  P427  Q444  A472  A480  T481  Y487  Q521  A542  I547  A551 | **Site**  161  180  187  191  197  200  213  249  264  295  346  362  390  408  414  416  423  426  432  450  464  536  540  543  560  565  610  612  636  642  656  720  761 | ***Ba***  M63  S80  E87  S91  G97  L100  D111  G128  Y143  R172  Q214  A227  K250  G258  A264  P266  T272  Q275  E281  Y295  D309  H349  T353  B356  N373  G378  R408  F410  R432  T438  Q452  G499  G514 | ***Hm***  R55  L72  N79  M83  L89  T92  N105  A133  H148  A178  A221  T234  E262  D271  P277  A279  V285  L288  K294  T312  K326  D378  A382  S385  L402  K407  L450  S452  Q476  A482  K496  V543  A556 |

**Sa:** *Sulfolobus acidocaldarius*

**Ba:** *Bacillus anthracis*

**Bh:** *Bacillus halodurans*

**Hm:** Human

| **Bacteria 1/ Archaea** | | | | | | | | **Bacteria 3/ Extremophile** | | | | | |
| --- | --- | --- | --- | --- | --- | --- | --- | --- | --- | --- | --- | --- | --- |
| **FD I**  **θI = 0.46±0.06**  **p = 0.50** | | | | **FD II**  **θII = 0.29±0.09**  **R = 3.0** | | | | **FD I**  **θI = 0.17±0.09**  **p = 0.47** | | | **FD II**  **θII = 0.08±0.11**  **R = 3.0** | | |
| **Site**  165  172  183  186  211  214  217  283  335  342  343  362  367  394  395  413  415  425  463  535  546  547  553  577  604  618  626  632  640  641  647  648 | ***Ec***  K67  A72  L83  T86  A110  F113  M116  R178  Q224  L231  E232  A248  Q253  S279  G280  S289  P291  V301  A335  E387  D398  K399  A405  N429  G455  V469  W476  P482  T490  V491  N497  S498 | ***Ta***  K31  I36  L47  T50  D74  G77  Q80  T126  L164  F192  R193  A209  A214  F240  T241  E250  S252  I261  D294  D327  D338  S339  S345  N369  M385  V399  Y406  M412  V420  Q421  E427  I428 | ***Sa***  E26  A31  L42  V45  A68  A71  W74  S140  Q185  L171  K172  M187  L192  K218  S219  E228  P230  L239  N273  R313  D324  G325  G330  N352  E365  L377  S385  A391  I399  H400  Y406  Y407 | **Site**  161  162  180  188  191  196  197  200  205  215  216  253  256  278  350  358  364  368  386  416  424  429  432  450  464  538  540  541  551  555  559  565  568  578  579  581  602  603  606  608  611  622 | ***Ec***  V63  D64  G80  P88  G91  G96  G97  L100  N105  R114  E115  T150  G153  A173  P239  T246  Q250  E254  A272  P292  I300  N305  E308  A322  D336  Q390  T392  H393  A403  T407  N411  G417  A420  Q430  M431  D433  A453  V454  N457  R459  S462  D472 | ***Ta***  R27  D28  S44  N52  C55  D60  L61  L64  N69  S78  G79  I112  S115  A135  F200  S207  I211  G215  P233  P253  V260  R265  E268  K281  K295  D330  T332  Y333  S343  I347  Y351  G357  V345  R370  G371  Y373  A383  L384  G387  R389  H392  E402 | ***Sa***  A22  K23  S39  P47  S50  D55  G56  L59  E64  S72  G73  L48  M101  A121  R179  K185  E189  G193  G211  P231  T238  K243  E246  R260  D274  D316  T318  F319  E328  I332  F336  G342  P360  R353  G354  G356  K363  P364  R367  R369  H372  K381 | **Site**  209  247  349  426  432  536  681 | ***Ba***  E107  H126  G217  Q275  E281  H349  I471 | ***Bh***  L82  P116  N215  E278  K284  R355  P483 | **Site**  180  196  213  215  216  338  346  540  541  555  558  559  561  612  632  636  637  640  720 | ***Ba***  S80  G96  D111  R113  E114  L206  Q214  T353  H354  T368  L371  S372  F374  F410  P428  R432  I433  I436  G499 | ***Bh***  A55  D71  N86  S88  G89  H204  A212  V359  Y360  I374  N377  Y378  G380  T420  M439  M443  Q444  G447  R511 |

**Ec:** *Escherichia coli*

**Ta:** *Thermoplasma acidophilum*

**Sa:** *Sulfolobus acidocaldarius*

**Ba:** *Bacillus anthracis*

**Bh:** *Bacillus halodurans*
