# Supplementary material for: “Phylogenetic and evolutionary analysis of functional divergence among Gamma glutamyl transpeptidase (GGT) subfamilies”
Source: Biol Direct. 2015 Sep 14;10:49. doi: 10.1186/s13062-015-0080-7 (PMC4568574; doi:10.1186/s13062-015-0080-7)

**Additional file 5**

**Figure S3: Phylogenetic tree of GGT proteins including non pathogenic proteobacteria.**

**
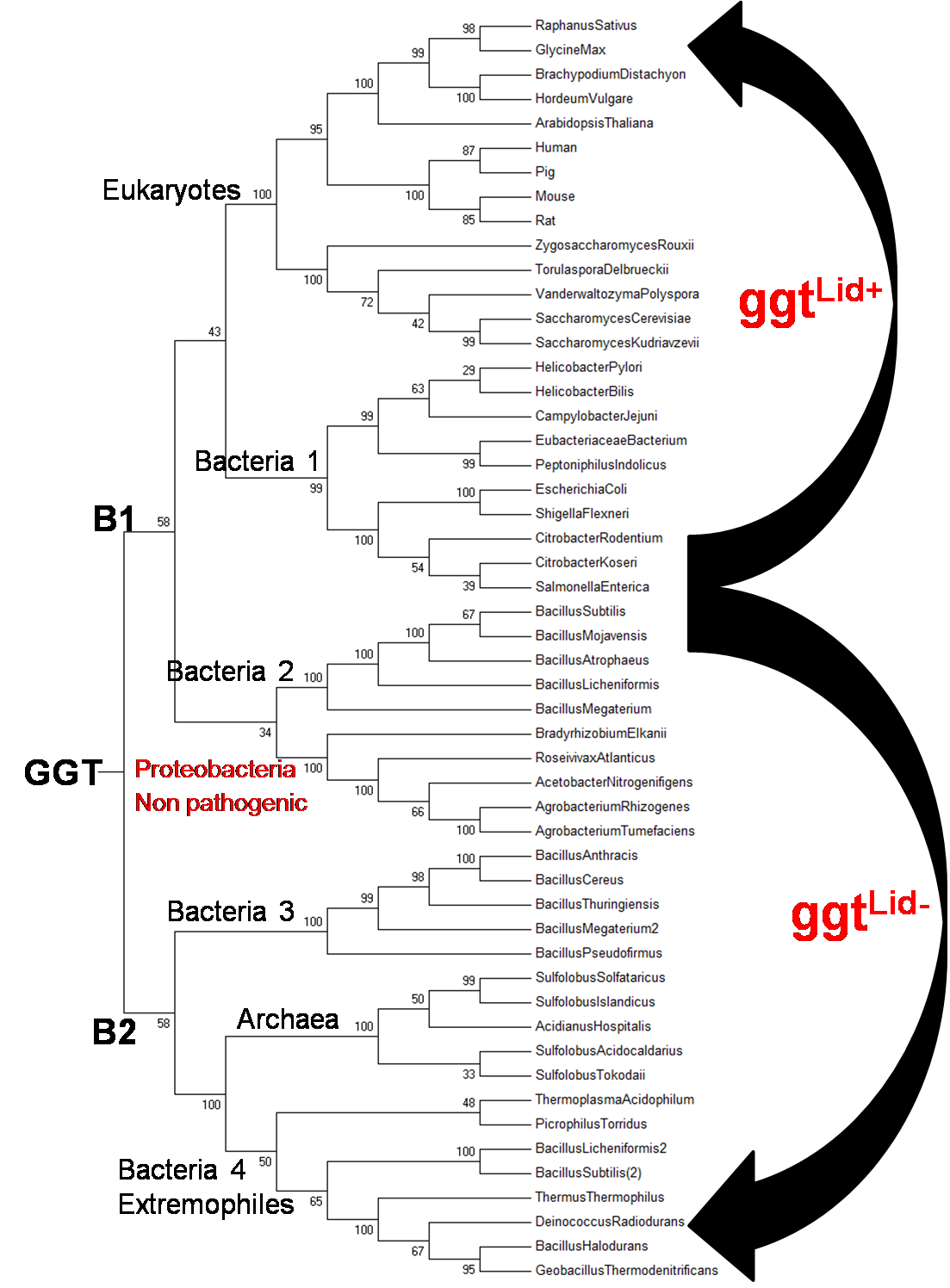
**

**Figure S4: Structure based sequence alignment of GGT proteins**

In the given figure, on the top of the row secondary structure elements of the GGT proteins have been shown with their respective numbering. GGT proteins are highly conserved in secondary structure pattern and shared αβ_1_β_2_α sandwich like protein folds. The alpha helical and β-strands regions of GGT proteins are represented by α and β notations respectively whereas remaing part of the aligned proteins might contain loop and coil regions. All secondary structure fragments are generated by using 3D structure information of *E. coli* GGT (2E0W). The 3D structure based sequence alignment is performed by using “TCoffee Expresso” server (<http://tcoffee.crg.cat/apps/tcoffee/do:expresso>) and final alignment figure is generated by using ESPript3.0 (http://espript.ibcp.fr/ESPript/ESPript/) online available tools. Highly conserved residues are hilighted in red color shadow.


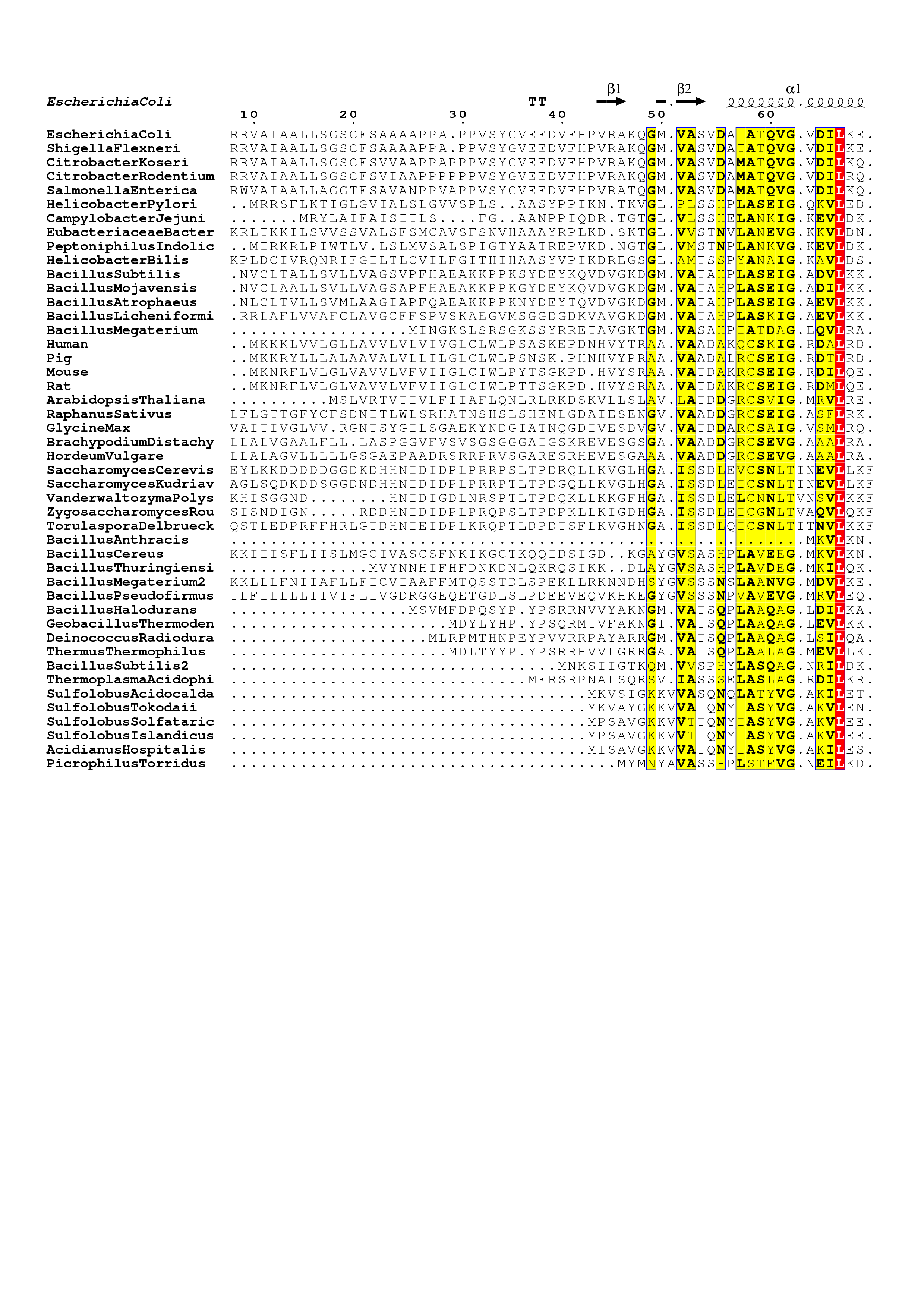


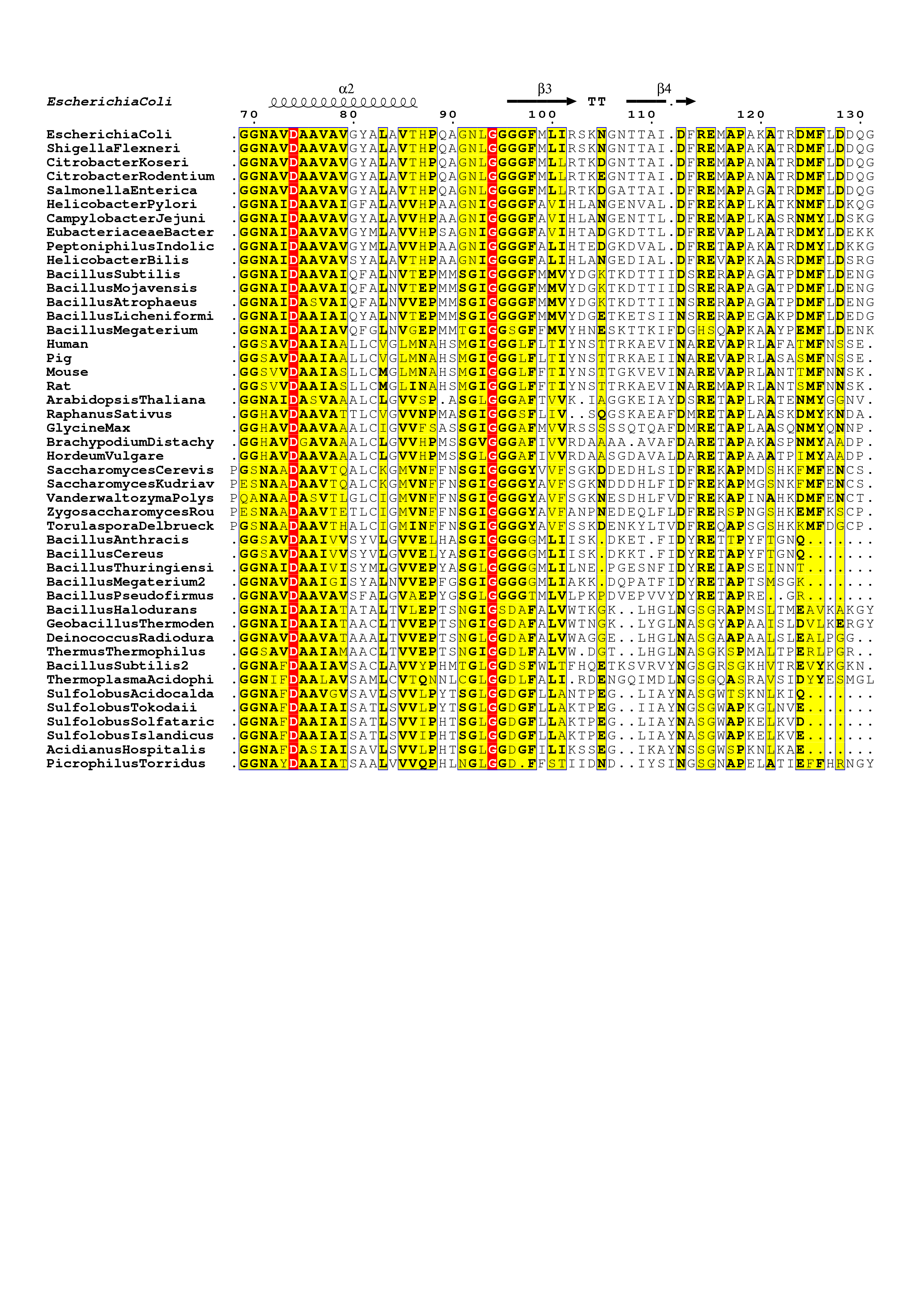


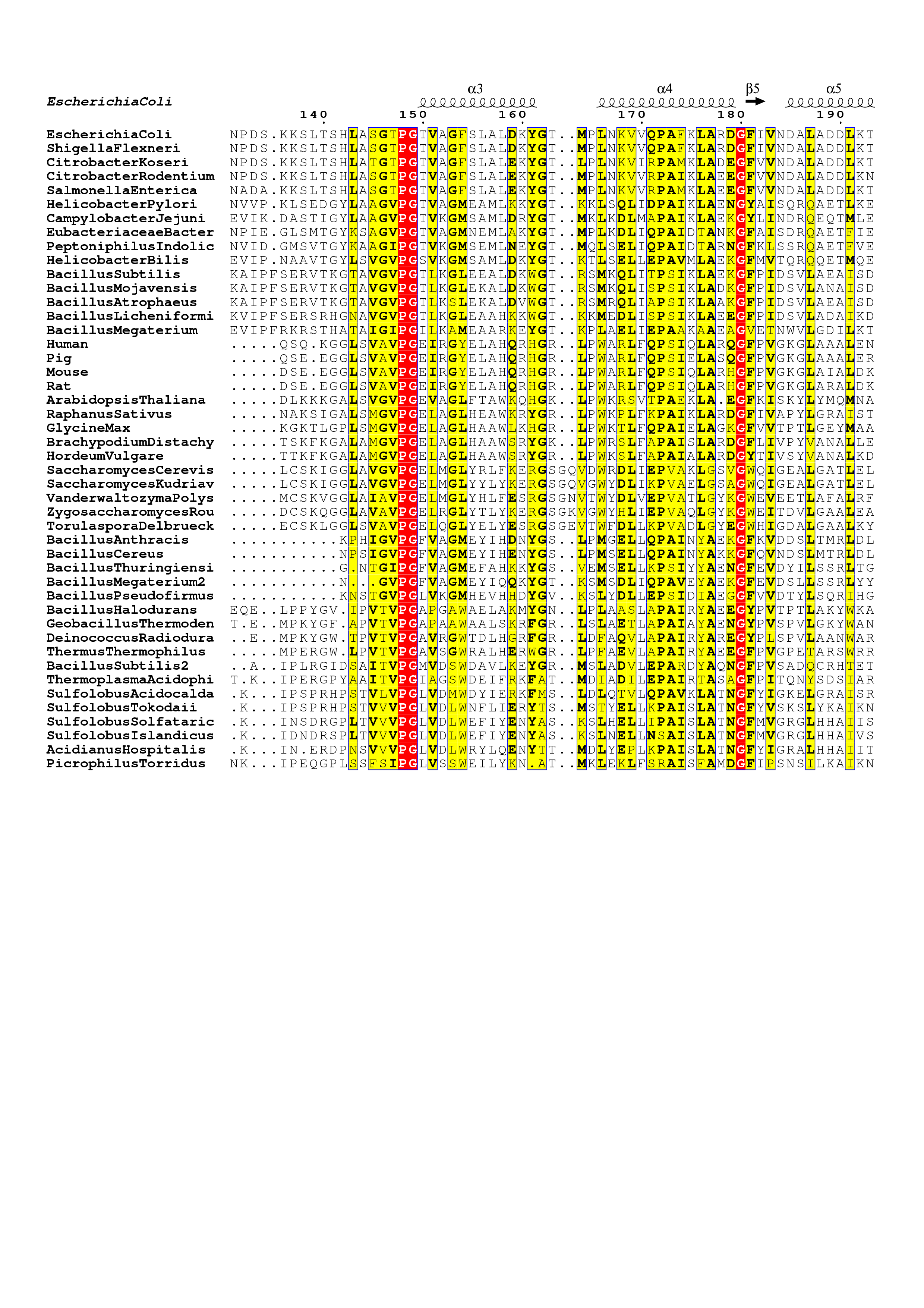


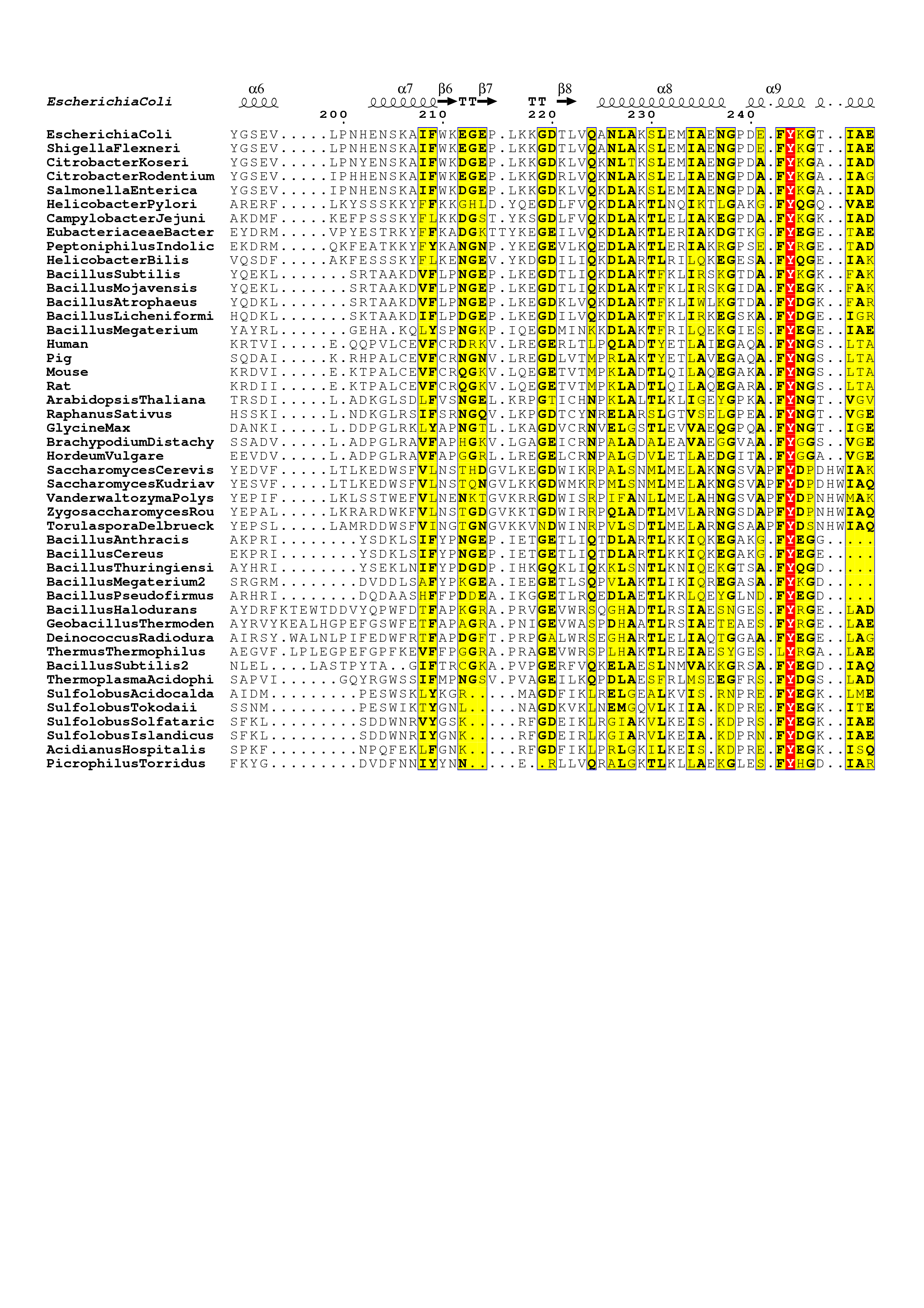


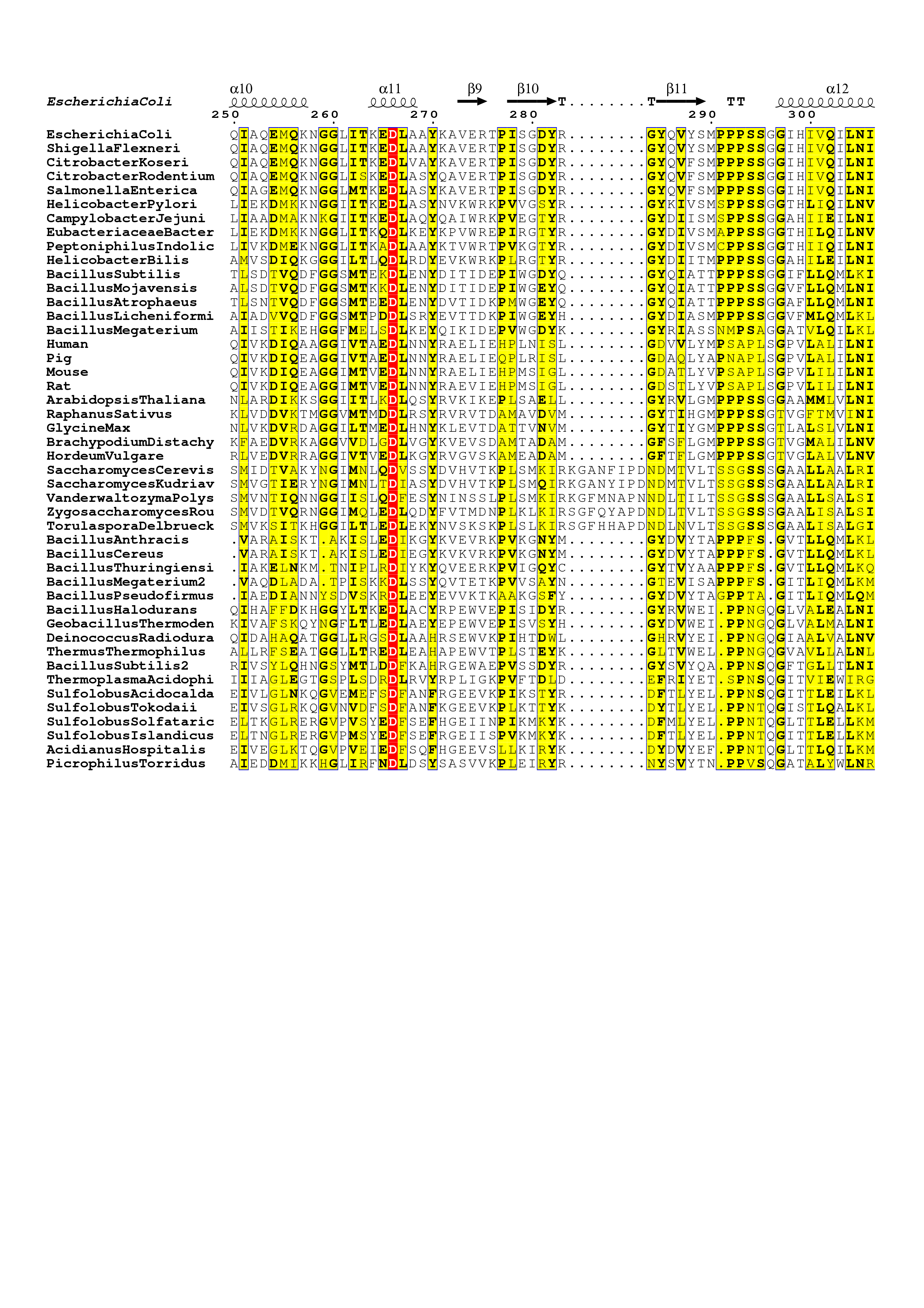


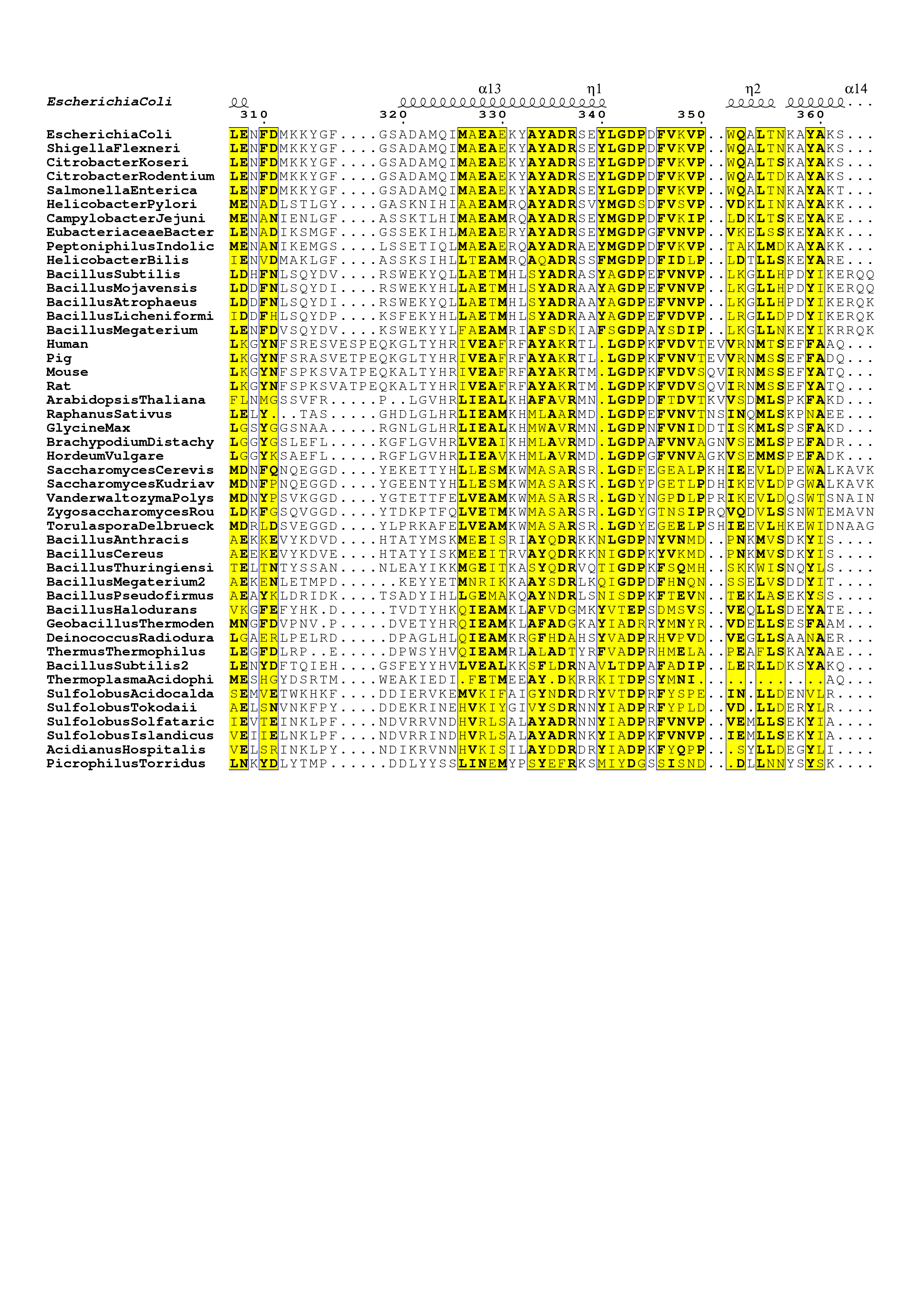


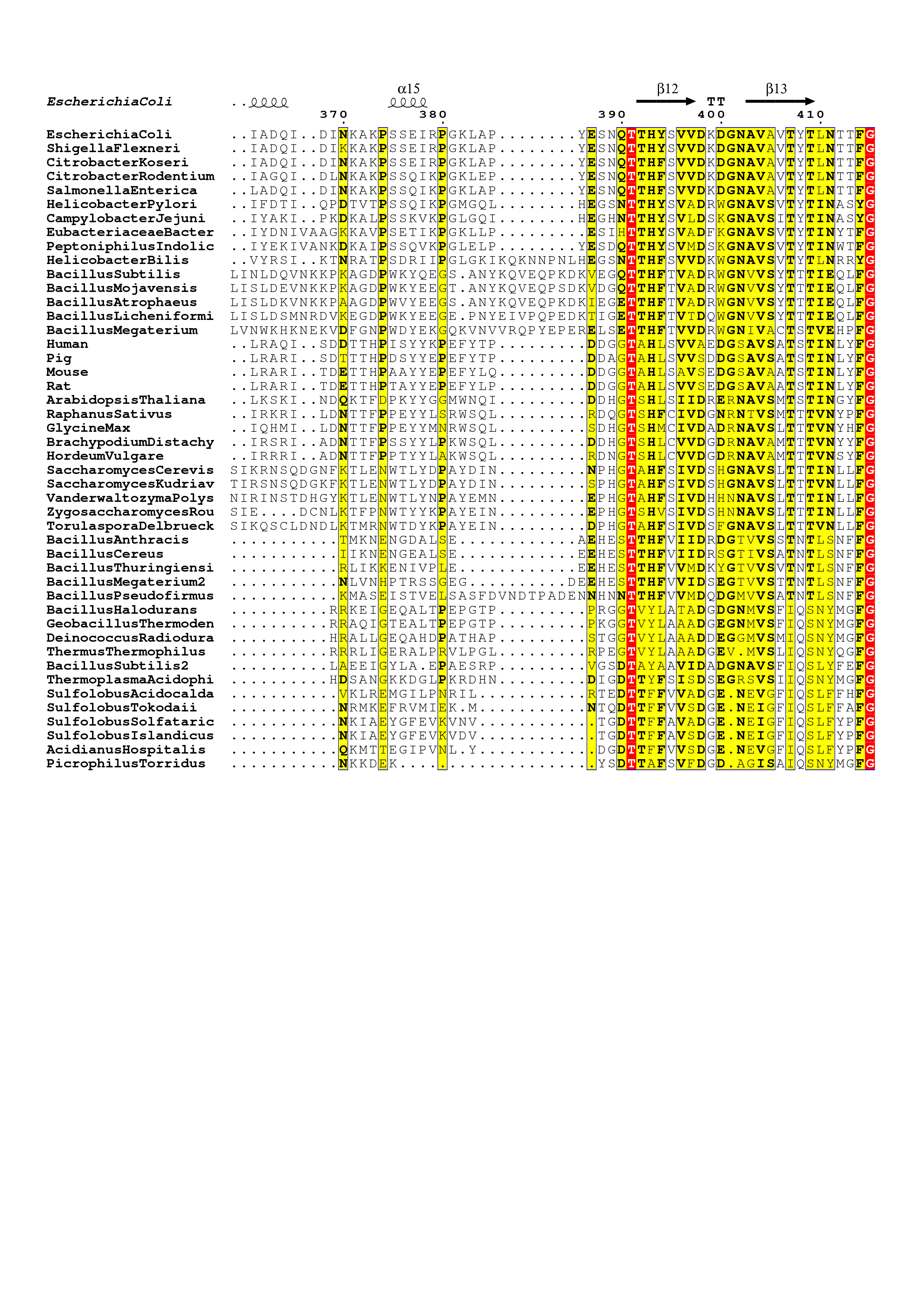


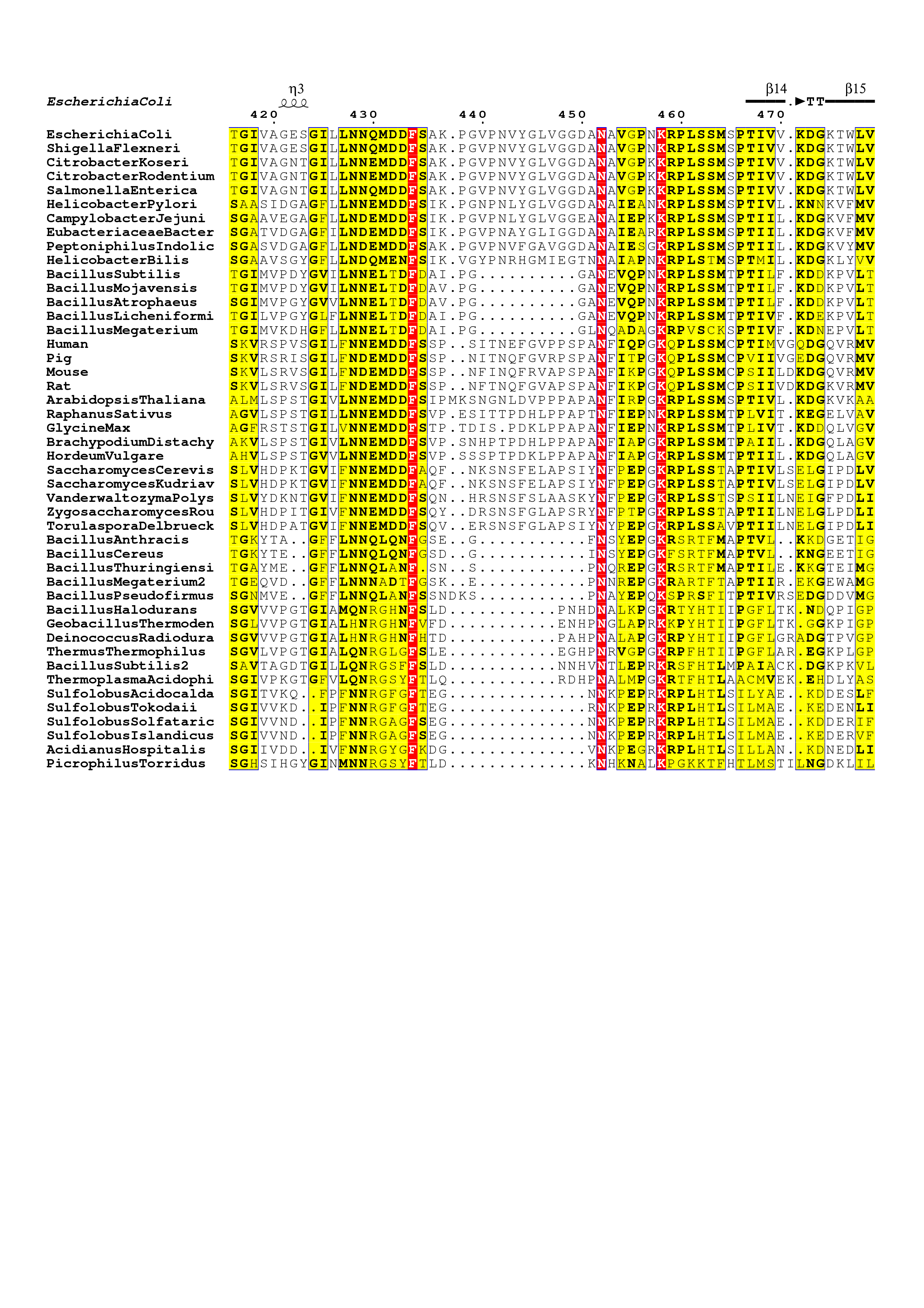


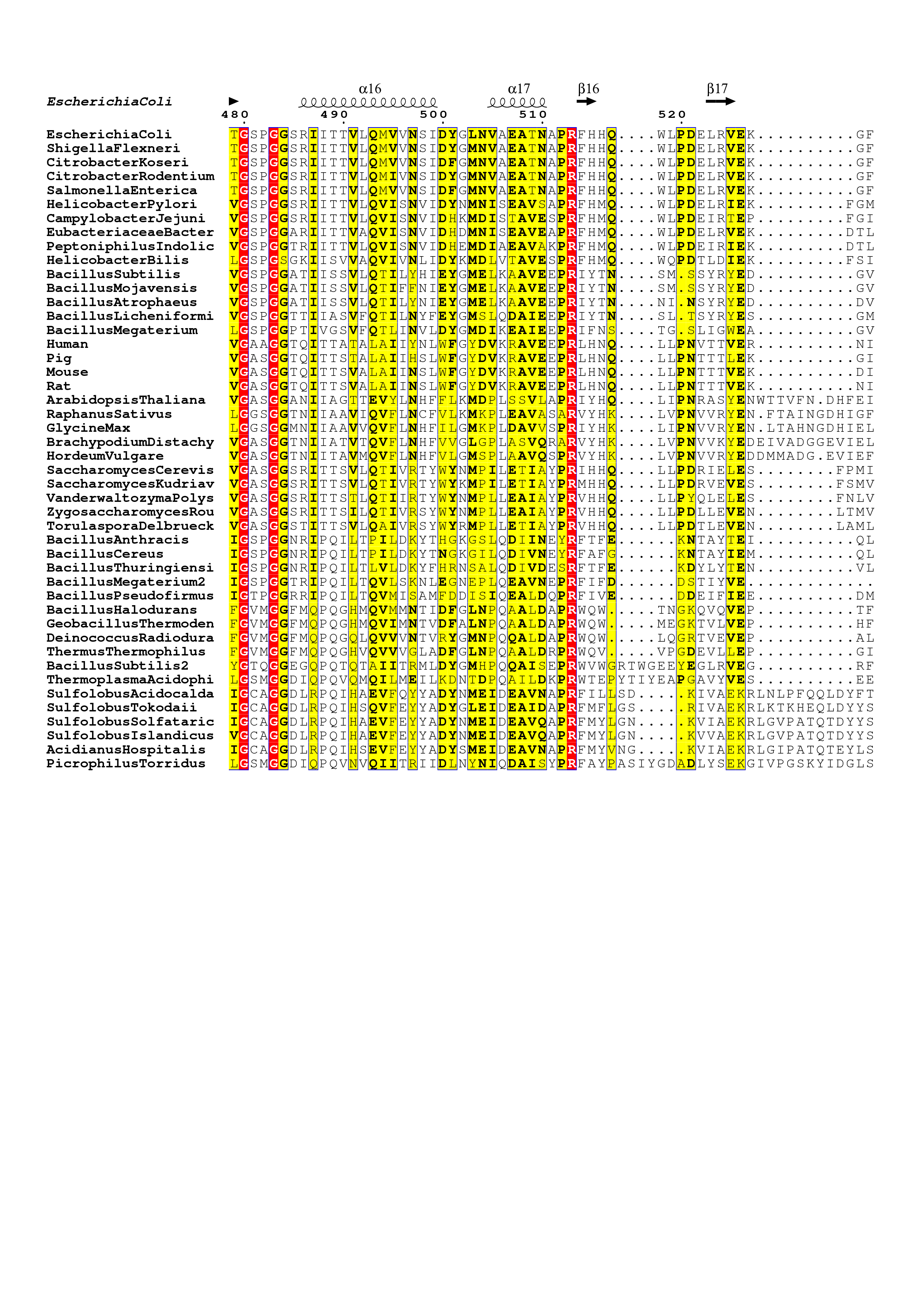


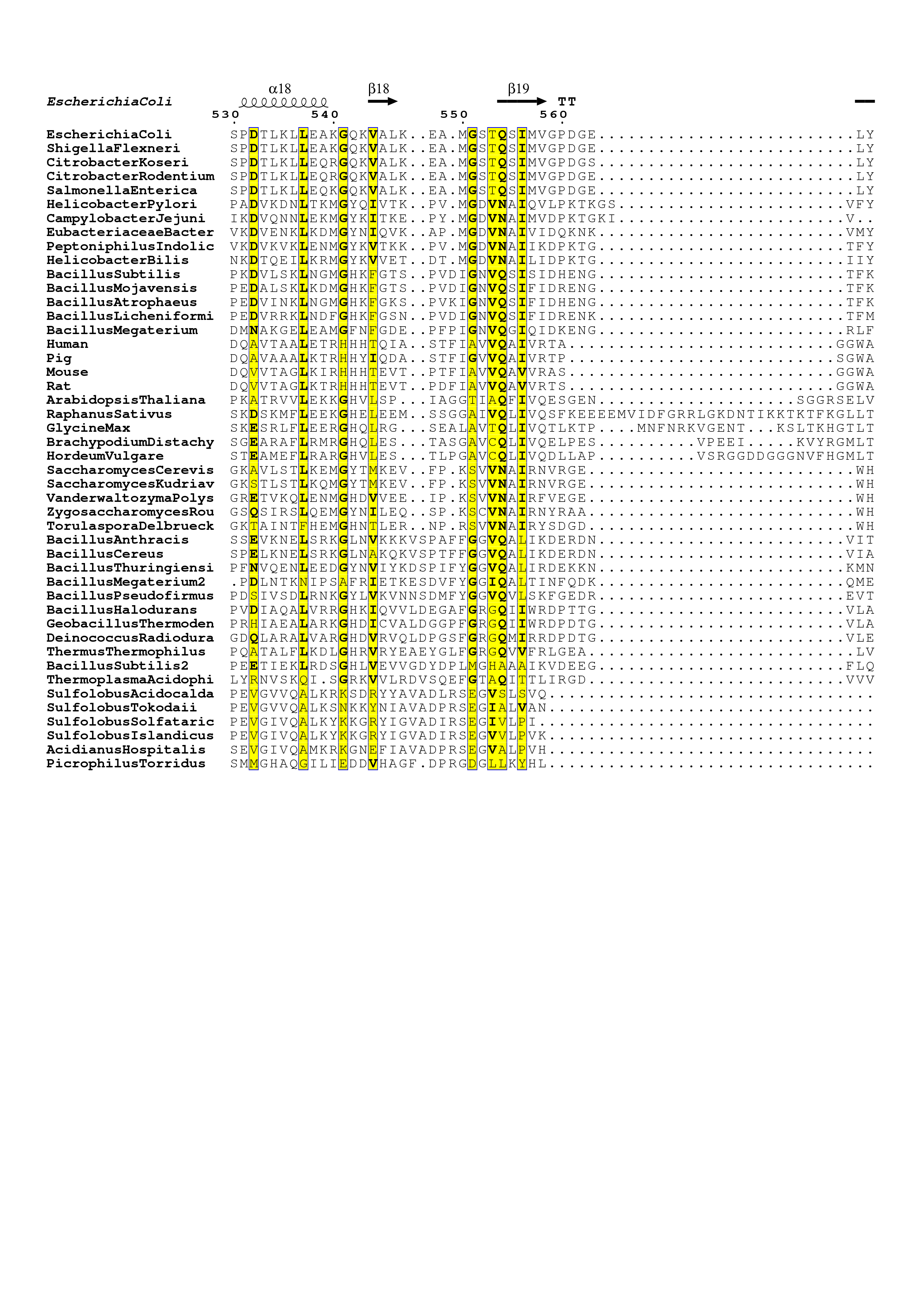


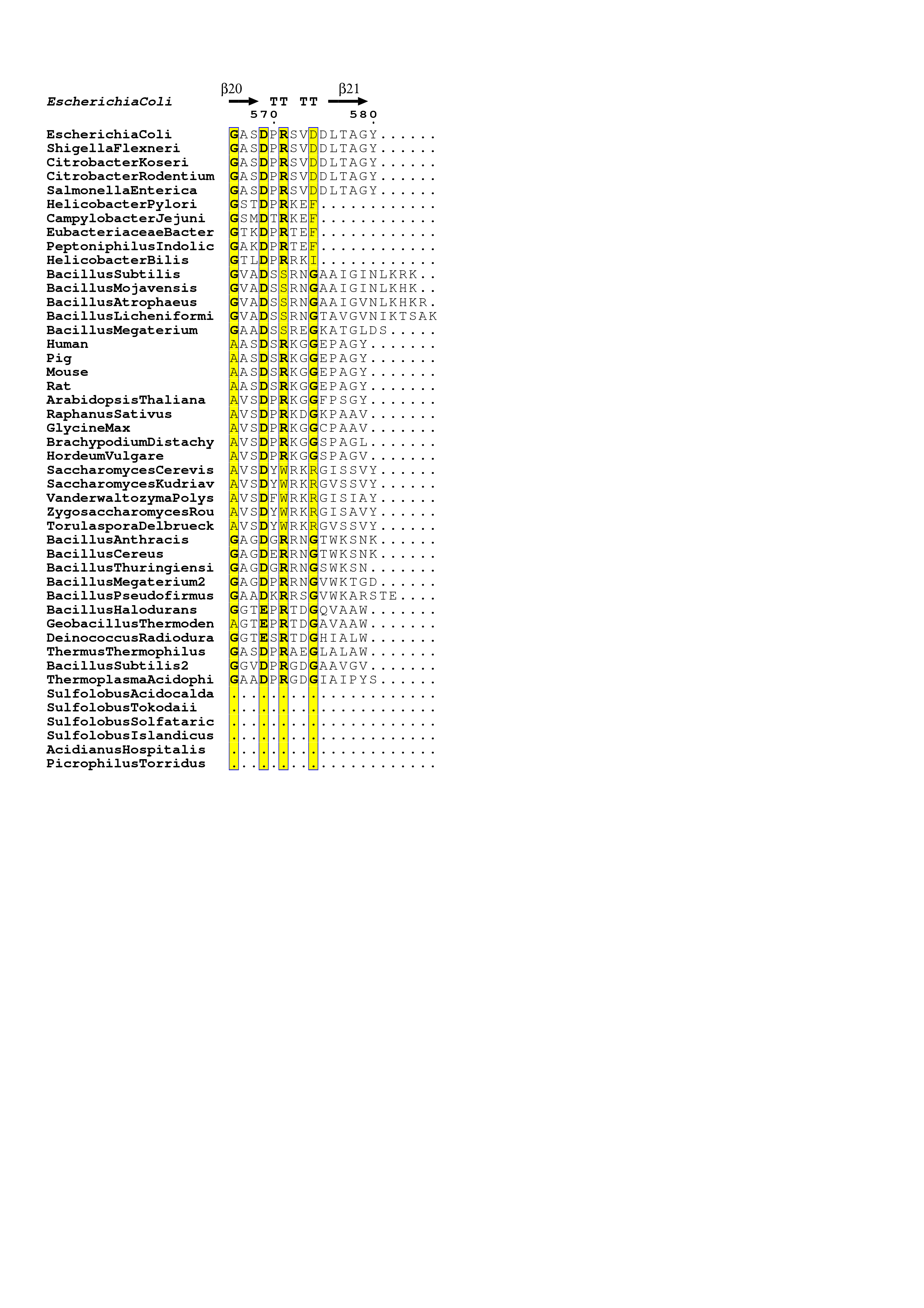

Supplement: Additional file 5: Figure S3. — Phylogenetic tree of GGT proteins including non pathogenic proteobacteria. Figure S4. Structure based sequence alignment of GGT proteins. In the given figure, on the top of the row secondary structure elements of the GGT proteins have been shown with their respective numbering. GGT proteins are highly conserved in secondary structure pattern and shared αβ1β2α sandwich like protein folds. The alpha helical and β-strands regions of GGT proteins are represented by α and β notations respectively whereas remaing part of the aligned proteins might contain loop and coil regions. All secondary structure fragments are generated by using 3D structure information of E. coli GGT (2E0W). The 3D structure based sequence alignment is performed by using “TCoffee Expresso” server (http://tcoffee.crg.cat/apps/tcoffee/do:expresso) and final alignment figure is generated by using ESPript3.0 (http://espript.ibcp.fr/ESPript/ESPript/) online available tools. Highly conserved residues are hilighted in red color shadow. (DOC 7750 kb) [file 13062_2015_80_MOESM5_ESM.doc]
